# HSV-1 employs UL56 to antagonise expression and function of cGAMP channels

**DOI:** 10.1016/j.celrep.2024.114122

**Published:** 2024-04-22

**Authors:** Henry TW Blest, Alexander Redmond, Jed Avissar, Jake Barker, Anne Bridgeman, Gerissa Fowler, Lise Chauveau, Jonny Hertzog, Iolanda Vendrell, Roman Fischer, Marie B Iversen, Lichen Jing, David M Koelle, Søren R Paludan, Benedikt M Kessler, Colin M Crump, Jan Rehwinkel

**Affiliations:** 1 Medical Research Council Human Immunology Unit, Medical Research Council Weatherall Institute of Molecular Medicine, Radcliffe Department of Medicine, University of Oxford, Oxford OX3 9DS, UK; 2 Department of Pathology, University of Cambridge, Cambridge CB2 1QP, UK; 3 Target Discovery Institute, Centre for Medicines Discovery, Nuffield Department of Medicine, University of Oxford, Oxford, UK; 4 Chinese Academy of Medical Sciences Oxford Institute, Nuffield Department of Medicine, University of Oxford, Oxford, UK; 5 Department of Biomedicine, Aarhus University, Aarhus C, Denmark; 6 Department of Medicine, University of Washington, Seattle, Washington, USA 98195; 7 Department of Laboratory Medicine and Pathology, University of Washington, Seattle, Washington, USA 98195; 8 Fred Hutchinson Cancer Center, Seattle, Washington, USA 98109; 9 Department of Global Health, University of Washington, Seattle, Washington, USA 98195; 10 Benaroya Research Institute, Seattle, Washington, USA 98101

**Keywords:** DNA sensing, cGAMP, cGAMP transport, VRAC, LRRC8A, SLC46A2, P2X7, herpes simplex virus, UL56

## Abstract

DNA sensing is important for anti-viral immunity. The DNA sensor cGAS synthesises 2’3’-cyclic-GMP-AMP (cGAMP), a second messenger that activates STING, which induces innate immunity. cGAMP not only activates STING in the cell where it is produced but also transfers to other cells. Transporters, channels and pores including SLC19A1, SLC46A2, P2X7, ABCC1 and volume-regulated anion channels (VRACs) release cGAMP into the extracellular space and/or import cGAMP. We report that infection with multiple human viruses depletes some of these cGAMP conduits. This includes herpes simplex virus 1 (HSV-1) that targets SLC46A2, P2X7 and the VRAC subunits LRRC8A and LRRC8C for degradation. The HSV-1 protein UL56 is necessary and sufficient for these effects that are mediated at least partially by proteasomal turnover. UL56 thereby inhibits cGAMP uptake via VRAC, SLC46A2 and P2X7. Taken together, HSV-1 antagonises inter-cellular cGAMP transfer. We propose this limits innate immunity by reducing cell-to-cell communication via the immunotransmitter cGAMP.

## Introduction

Pattern recognition receptor (PRR) signalling is an essential initiating event for innate and adaptive immune responses, including during virus infection ^1^. Agonists known as pathogen- or danger-associated molecular patterns (PAMPs/DAMPs) engage PRRs that then relay the signal to adaptor proteins. These in turn mediate the induction of downstream signalling and effector functions. For example, PRR signalling can result in gene transcription, including of genes encoding cytokines, or in cytokine maturation. Typically, these signalling cascades occur intracellularly and in a cell-autonomous manner, whereby individual cells execute all steps from signal detection to activation of an effector function.

One PRR that attracted much interest in recent years is cGAS ^2^. cGAS is a sensor for dsDNA and to a lesser extent DNA:RNA hybrids ^2,3^. Through sensing the intracellular accumulation of unusual nucleic acids, cGAS detects infections with many pathogens. cGAS also initiates and/or amplifies inflammatory responses in cancer, neurodegeneration, myocardial infarction, ageing and obesity, amongst other conditions, with beneficial and detrimental consequences in different disease settings ^4^. Unlike other PRR signalling pathways, cGAS does not engage its adaptor protein STING through direct protein-protein interactions. Instead, DNA binding activates cGAS’ catalytic activity to synthesise the dinucleotide 2’3’ cyclic GMP-AMP (cGAMP). cGAMP is a small, polar and diffusible molecule that then binds to STING, which in turn activates downstream signalling ^5^.

Interestingly, cGAMP not only activates STING in the cell where it is synthesised by cGAS. cGAMP is also transferred to other cells, where it induces STING-dependent but cGAS-independent signalling. This expands on the principle of cell-autonomous PRR signalling and serves to propagate the response to cells with inactive cGAS, such as uninfected bystander cells in the context of viral infection. cGAMP is transferred between cells by different mechanisms. These include diffusion through gap junctions, which allows for the activation of STING in cells located adjacent to a cell with active cGAS ^6^. Another mechanism of cell-to-cell transfer of cGAMP is its inclusion in virus particles or enveloped vesicles ^7,8^, which can be exploited for vaccination and induction of anti-tumour immunity ^9,10^. In these settings, cGAMP remains topologically ‘inside’ cells.

Work published over the last four years revealed that cGAMP is also released into the extracellular space and is imported from the outside into cells. As such, cGAMP has been referred to as an ‘immunotransmitter’ ^11–13^. At least five different transporters, channels and pores mediate cGAMP export and/or import. These include: (1) SLC19A1 ^14,15^, a folate transporter also known as reduced folate carrier 1 (RFC1); (2) the poorly characterised SLC46A family ^15,16^; (3) P2X7 ^17^, an ATP-gated, non-selective pore for hydrophilic substances of up to ∼0.9 KDa; (4) volume-regulated anion channels (VRACs) ^18,19^, heterohexameric channels that open in response to osmotic stress; and (5) ABCC1 ^20^, an ABC transporter. VRAC and ABCC1 have been reported to mediate export of cGAMP from the intracellular environment across the plasma membrane into the extracellular space. VRAC can also allow for import of cGAMP into cells, as do SLC19A1, the SLC46A family and P2X7.

Emerging evidence suggests these proteins are important for immune responses. For example, ABCC1 controls disease severity in a model of autoimmunity ^20^, P2X7 facilitates anti-cancer immunity ^17^ and VRAC channels containing the LRRC8C subunit suppress T cell function^21^. In addition, in a mouse model of herpes simplex virus 1 (HSV-1) infection, LRRC8A/E containing VRAC channels play an antiviral role ^18^. The importance of extracellular cGAMP in host defence against HSV-1 is further underscored by enhanced resistance of mice expressing a catalytically inactive version of ENPP1, an extracellular cGAMP hydrolase ^22,23^. Notwithstanding these interesting results, little is known about the roles of the different cGAMP transporters, channels and pores in different viral infections, and why such a large and diverse group of proteins is involved in cGAMP transfer between cells.

Viruses have evolved a myriad of strategies to inhibit PRR signalling ^24^. Large DNA viruses typically encode multiple antagonists, including proteins that target the cGAS-STING pathway ^25^. However, with the notable exception of a poxviral cGAMP nuclease ^26^, viruses are thus far not known to directly target cGAMP. Additionally, to date no viral antagonist of cGAMP transport has been described. Here, we reasoned that viruses are likely to have developed means of antagonising cGAMP transporters, channels or pores and set out to identify such mechanisms. We screened a panel of viruses representing major viral families for their ability to downregulate the protein abundance of known cGAMP conduits. We found that multiple viruses impacted protein levels of LRRC8A, LRRC8C, P2X7 and/or SLC46A2. Functional studies using HSV-1 revealed that the viral UL56 protein mediated degradation of these cGAMP conduits in infected cells. UL56 thereby limited the capacity for type I interferon (IFN) induction triggered by extracellular cGAMP.

## Results

### Screening for viral antagonism of cGAMP transporters, channels and pores

**Figure 1:**
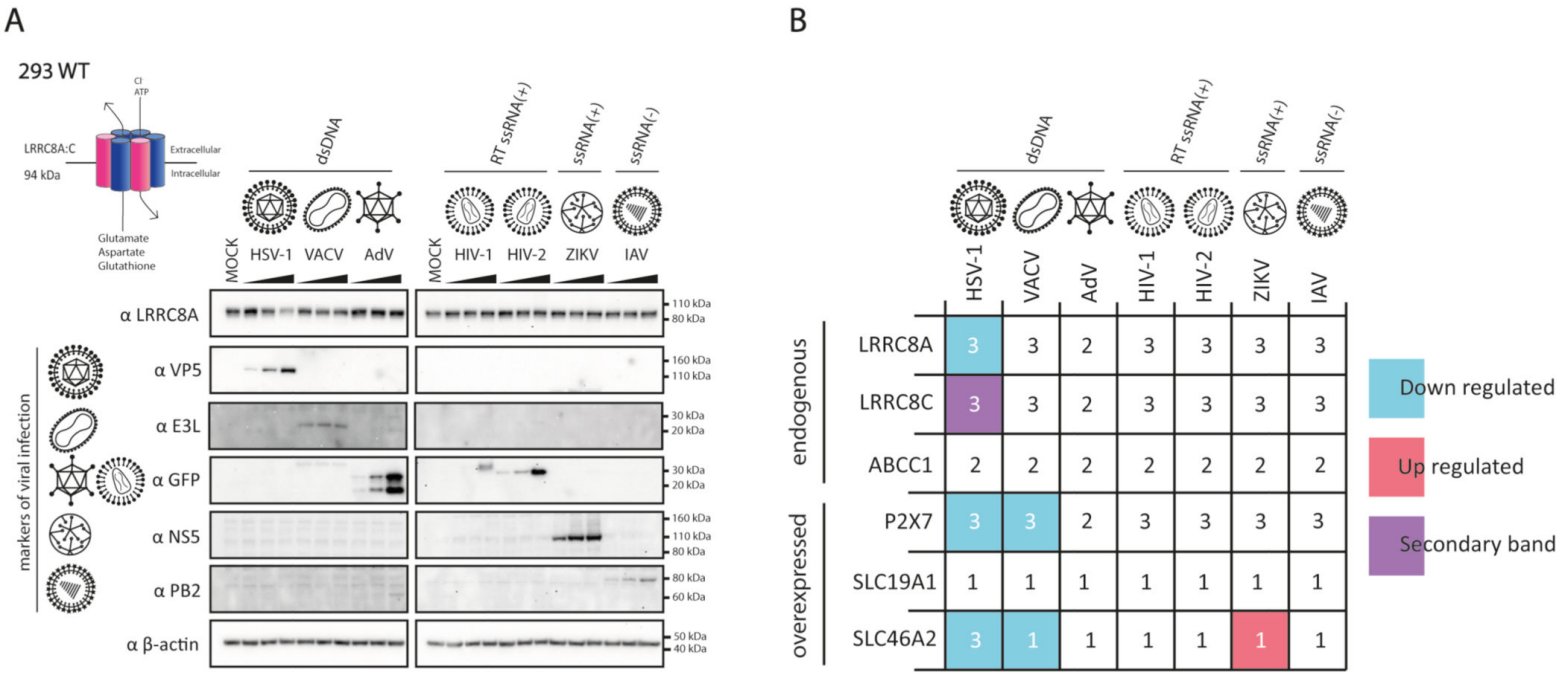
Viral infection affects protein levels of cGAMP channels, transporters and pores. (A) HEK293 cells were infected with HSV-1 (MOI – 0.02, 0.006, 0.002), VACV (MOI – 0.2, 0.066, 0.022), AdV (MOI – 1500, 150, 15), HIV-1 (1:10, 1:50, 1:250), HIV-2 (1:10, 1:50, 1:250), ZIKV (MOI – 1, 0.25, 0.06) and IAV (MOI – 10, 5, 2.5). 48 hours later, cells were lysed, and abundance of the indicated proteins was assessed by western blot. β-Actin served as a loading control. (B) Summary of the protein levels of cGAMP conduits following infection with different viruses. Data in (A) are representative of two (AdV) and three biological repeats (all other viruses) and the numbers provided in (B) indicate the number of biological repeats for each protein tested for the indicated infections. See also Figures S1-S3.

Given the importance of VRAC and ENPP1 in host defense against HSV-1 ^18,22^, we surmised that this and other viruses may interfere with the expression of cGAMP transporters, channels and pores. To test this idea, we infected the human cell line HEK293 with a panel of viruses. We included three DNA viruses, namely HSV-1 (KOS strain), vaccinia virus (VACV, WR strain) and human adenovirus type 5 (AdV, AdV-Cre-GFP strain), two retroviral vectors (single round, VSV-G pseudotyped HIV-1 and HIV-2 expressing GFP) and two RNA viruses, Zika virus (ZIKV, isolate ZIKV/*H. sapiens*/Brazil/PE243/2015) and influenza A virus (IAV, PR8 strain). 48 hours after infection using different multiplicities of infection (MOIs), we prepared cell lysates for western blot analysis. We chose this late timepoint to capture effects that happen during all stages of viral life cycles or upon viral spread. We validated that cells were infected by using antibodies detecting virally expressed proteins such as the HSV-1 capsid protein VP5. As expected, VP5, E3L, NS5 and PB2 proteins were detected in cells infected with HSV-1, VACV, ZIKV and IAV, respectively, with signal intensities depending on viral dose (Figure 1A). AdV infection and HIV-1 and HIV-2 transduction were confirmed by expression of GFP. Next, we probed for the VRAC subunits LRRC8A and LRRC8C. HSV-1 infection led to a dose-dependent decrease in abundance of LRRC8A (Figure 1A). At the highest MOI tested, we detected a faster migrating band with an α-LRRC8C antibody in HSV-1 infected cells, potentially indicating protein cleavage (Figure S1). The LRRC8A and LRRC8C western blot signals were unchanged upon infection with all other viruses tested.

We were unable to detect P2X7, SLC19A1 and SLC46A2 using commercially available antibodies in lysates from HEK293 cells; this may be due to lack of expression in this cell line or to low antibody specificity. We therefore stably transduced HEK293 cells with lentiviral constructs expressing C-terminally V5-tagged P2X7, SLC46A2 or SLC19A1. Using an α-V5 antibody, we detected P2X7-V5 by western blot at the expected molecular weight (Figure S2A). However, for both SLC19A1 and SLC46A2, slowly migrating smears were apparent. Based on western blot protocols for transmembrane proteins ^27^, we omitted boiling samples prior to gel loading and incubated samples instead on ice or at 37°C. In both settings, signals at the expected molecular weight were detected for SLC19A1 and SLC46A2 (Figures S2B and S2C). Therefore, in subsequent experiments, samples were simply kept on ice before loading with urea added as a denaturing reagent. Next, as described above, we infected the stably transduced cell lines and analysed lysates by western blot. The levels of P2X7-V5 were reduced in cells infected with HSV-1 and VACV (Figure S2D). SLC19A1-V5 levels were unchanged in cells infected with all viruses tested (Figure S2E) and SLC46A2-V5 abundance was modulated by HSV-1, VACV and ZIKV infections (Figure S2F). Finally, we tested ABCC1. Again, detection by western blot was aided by omitting sample boiling (Figure S3A). We found that protein levels of endogenous ABCC1 were unchanged upon virus infection in our setting (Figure S3B). Taken together, our data – summarised in Figure 1B, with quantification provided in Figure S3C – show that viral infections result in altered levels of at least three cGAMP conduits and therefore provide a resource for future studies. Our results further suggest that HSV-1 may be particularly adept at downregulating the abundance of these proteins.

### HSV-1 infection reduces abundance of VRAC subunits

To validate and explore the functional implications of our findings, we next focussed on HSV-1’s impact on expression of VRAC, which promotes innate immune responses to this virus ^18^. VRAC channels transport different anionic substrates along concentration gradients and are activated under hypotonic conditions ^28^. In a process called regulatory volume decrease (RVD), VRACs release osmolytes to prevent cell swelling during osmotic stress. VRACs are hexamers composed of LRRC8 subunits. Humans have five *LRRC8* genes (*A-E*). LRRC8A (also known as SWELL1) is an obligatory constituent of VRAC channels and pairs with LRRC8B-E subunits. cGAMP is transported by LRRC8A:C/E channels ^18,19^.

**Figure 2:**
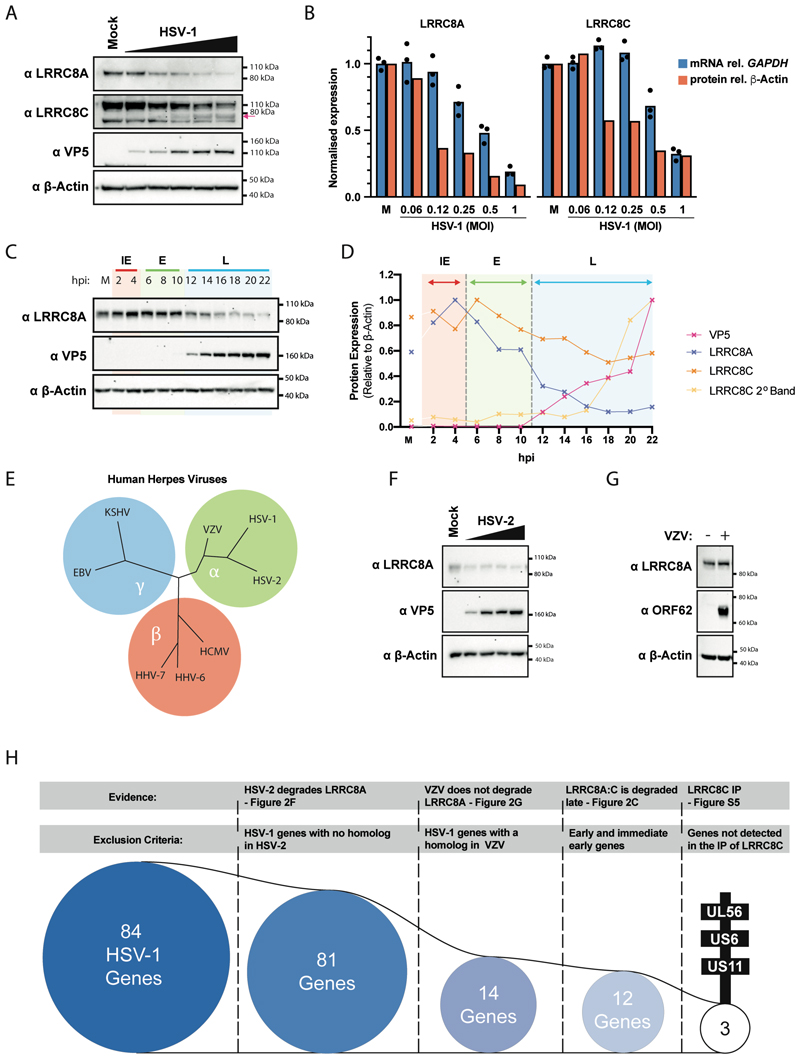
HSV-1 targets VRAC. (A) HEK293 cells were infected with HSV-1 (MOI = 0.06, 0.12, 0.25, 0.5 and 1). After 24 hours, cells were lysed, and the indicated proteins were detected by western blot. β-Actin served as a loading control. The arrow indicates a faster migrating band detected by the α-LRRC8C antibody. (B) Total RNA was extracted from cells infected in (A). *LRRC8A* and *LRRC8C* mRNA levels were determined by RT-qPCR and normalised to *GAPDH* mRNA. The western blot in (A) was quantified by densitometry. Data were set to 1 in mock infected cells (M). (C, D) HEK293 cells were infected with HSV-1 (MOI = 1) and lysed at the indicated time points. Samples were analysed as in (A) by western blot (C), which was quantified by densitometry (D). Data for LRRC8C are from Figure S4A. (E) Schematic showing phylogeny of human herpes viruses. (F) HEK293T cells were infected with HSV-2 (MOI = 1, 3, 5 and 10). After 24 hours, cells were lysed, and the indicated proteins were detected by western blot. (G) VZV-infected MeWo cells were co-cultured with uninfected MeWo cells at a ratio of 1:5 (infected : uninfected) for one hour. Infected inoculum cells were then washed off and cells were incubated for 24 hours. The indicated proteins were detected by western blot in cell lysates. (H) Overview showing how three candidate genes were identified amongst the 84 genes encoded by HSV-1. Data in (A), (B) and (G) are representative of three biological repeats. In (B), data points show technical triplicates of the RT-qPCR. Data in (C), (D) and (F) are representative of two biological repeats. See also Figures S4 and S5 and Tables S1 and S2.

In line with the results from our screen (Figure 1), infection of HEK293 cells with increasing MOIs of HSV-1 led to reduced protein levels of the VRAC subunit LRRC8A (Figure 2A). This effect was less pronounced but nonetheless reproducible for LRRC8C (Figure 2A). For this subunit, a band migrating at about 80 kDa was apparent at higher viral doses. HSV-1 non-specifically prevents the production of many cellular proteins in a process called host cell shutoff, which leads to reduced levels of messenger RNAs (mRNAs) ^29^. To test whether the loss of VRAC subunits in HSV-1-infected cells was simply due to host cell shutoff, we concurrently analysed LRRC8A and LRRC8C mRNA and protein levels. At high doses of HSV-1, mRNA and protein levels were reduced for both subunits (Figure 2B). However, at lower MOIs (0.12-0.25), HSV-1 infection had little or no impact on *LRRC8A* and *LRRC8C* mRNA levels, whilst protein abundance was reduced (Figure 2B). This suggested that mechanisms other than host cell shutoff contributed to the loss of LRRC8A and LRRC8C proteins from infected cells. To further characterise this process, we analysed VRAC subunit levels over time following HSV-1 infection. Reduced abundance of LRRC8A and LRRC8C was first detected at 12 hours and 16 hours after infection, respectively (Figures 2C, 2D and S4A). This suggested that loss of VRAC subunits occurred when HSV-1 genes belonging to the late kinetic class such as VP5 were expressed. We also noted that HSV-1 infection did not downregulate the level of mouse LRRC8A in immortalised mouse embryonic fibroblasts (Figure S4B).

### HSV-1 UL56 targets LRRC8A and LRRC8C for degradation

We next hypothesised that an HSV-1-encoded protein targets VRAC subunits for degradation. To identify such a factor, we analysed LRRC8A levels in cells infected with the closely related α-herpesvirus HSV-2 (Figure 2E). Like HSV-1 infection, HSV-2 infection in HEK293T cells led to reduced abundance of LRRC8A (Figure 2F). However, Varicella-Zoster virus (VZV) -infected human MeWo cells showed no reduction in protein levels of LRRC8A and LRRC8C (Figures 2G and S4C). This indicated that a viral protein encoded in the HSV-1 and HSV-2 genomes, but not by the more distantly related α-herpesvirus VZV, targeted VRAC subunits. These criteria narrowed our search to 14 HSV-1 genes, of which twelve were ‘late’ genes (Figure 2H and Table S1). In parallel, we attempted to identify viral proteins targeting VRAC by proteomics. We generated HEK293 cells stably expressing LRRC8A-V5 or LRRC8C-V5. Following HSV-1 infection, we used an α-V5 antibody to immunoprecipitate VRAC and associated proteins. Western blot analysis confirmed successful precipitation of both subunits (Figures S5A and S5B). Silver staining revealed that several other proteins were associated with LRRC8C, particularly after viral infection (Figure S5B). We used mass spectrometry to identify these proteins (Figure S5C and Table S2). As expected, LRRC8C-derived peptides were highly abundant in LRRC8C precipitates from uninfected and infected cells. We also identified three other VRAC subunits: LRRC8A, LRRC8D and LRRC8E (Figure S5C). This validated our immunoprecipitation approach. Several HSV-1 proteins co-precipitated with LRRC8C from infected cells. Intersection of LRRC8C-associated HSV-1 proteins above the abundance limit (Figure S5C) with the set of twelve genes described earlier further narrowed our list of candidate factors to UL56, US6 and US11 (Figures 2H and S5C and Table S1).

**Figure 3:**
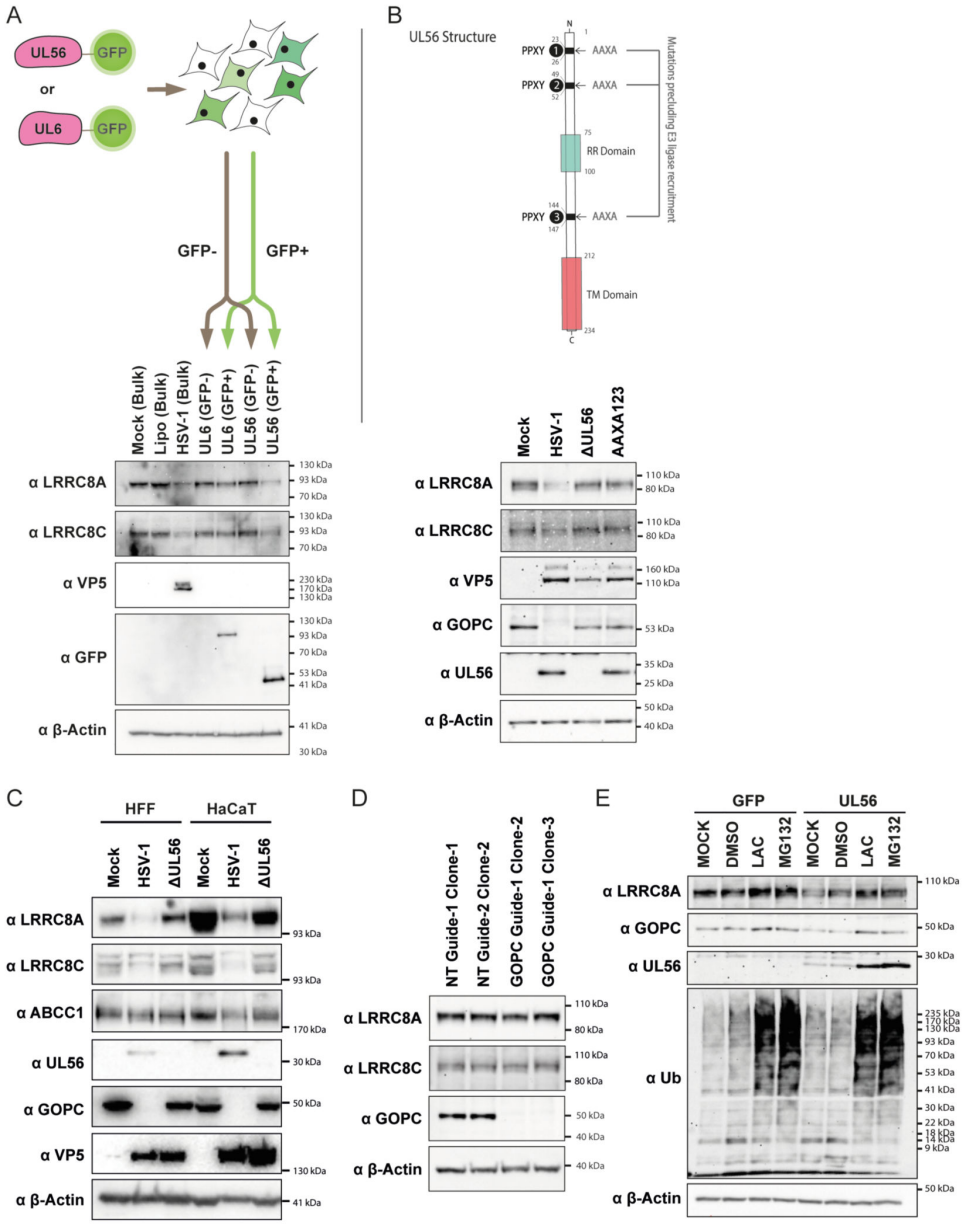
UL56 is necessary and sufficient for LRRC8A and LRRC8C degradation. (A) HEK293T cells were transiently transfected with expression plasmids encoding UL56-GFP or UL6-GFP. 24 hours later, GFP- and GFP+ cells were enriched by FACS. The gating strategy is shown in Figure S6. Untreated cells (Mock), lipofectamine treated cells (Lipo) and HSV-1 infected cells (MOI = 3, 24 hours) were included as controls without sorting. The experimental strategy is shown (top) and samples were analysed by western blot using the indicated antibodies (bottom). β-Actin served as a loading control. (B) Motifs and domains found in UL56 are shown (top). RR, arginine-rich region; TM, transmembrane domain. HEK293 cells were infected with HSV-1 of the indicated genotypes (MOI = 3, 24 hours). Cell lysates were analysed by western blot. (C) HFFs or HaCaT cells were infected with HSV-1 of the indicated genotypes (MOI = 1, 48 hours). Cell lysates were analysed by western blot. (D) Gene targeting in HEK293 cells was performed using CRISPR-Cas9 with non-targeting (NT) or *GOPC*-targeting sgRNAs. Clonal cell lines were obtained and analysed by immunoblot. (E) HEK293T cells stably transduced with GFP or UL56 were treated with either DMSO, lactacystin (20 μM) or MG132 (5 μM) for 24 hours. Cell lysates were then analysed by western blot. Data are representative of three biological repeats. See also Figure S6.

Amongst these viral proteins, UL56 is known to target multiple cellular proteins for proteasomal degradation ^30^. We therefore focussed on UL56. To test whether UL56 was able to target VRAC subunits, we overexpressed a UL56-GFP fusion protein in HEK293T cells by transient transfection. UL6-GFP served as a negative control. Using fluorescence-activated cell sorting (FACS), we isolated GFP+ and GFP- cells for analysis by western blot (Figures 3A and S6). LRRC8A and LRRC8C abundance was reduced in cells expressing UL56-GFP, but not in cells with UL6-GFP, suggesting that UL56 was sufficient to mediate a reduction in protein levels of these two VRAC subunits (Figure 3A). Next, we infected HEK293 cells with a virus unable to express UL56 (HSV-1 ΔUL56) ^30^. LRRC8A and LRRC8C protein levels were unchanged in cells infected with HSV-1 ΔUL56 (Figure 3B). UL56 contains three PPXY motifs that recruit NEDD4 family E3 ubiquitin ligases ^30^. Mutation of the PPXY motifs to AAXA prevents the recruitment of these ligases. We found that LRRC8A and LRRC8C protein levels were normal in cells infected with HSV-1 encoding UL56-AAXA123, which bears AAXA mutations in all three motifs (Figure 3B). To expand our observations to primary cells and cell types naturally infected by HSV-1, we used primary human foreskin fibroblasts (HFFs) and the keratinocyte cell line HaCaT (Figure 3C). Much like in HEK293 cells, HSV-1 infection reduced protein levels of LRRC8A and LRRC8C, whereas ABCC1 was unaffected. Moreover, HSV-1 ΔUL56 failed to target LRRC8A and LRRC8C (Figure 3C). UL56 is therefore necessary and sufficient for the reduced levels of VRAC subunits observed in HSV-1 infected cells.

One known protein targeted by UL56 for proteasomal degradation is the cellular trafficking factor Golgi-associated PDZ and coiled-coil motif-containing protein (GOPC), which is required for the transport of other proteins such as TLR2 to the cell surface ^30^. It was therefore conceivable that reduced levels of LRRC8A and LRRC8C were due to a trafficking defect, indirectly mediated by UL56 via GOPC degradation. To test this, we generated *GOPC* knockout HEK293 cells using CRISPR-Cas9 (Figure 3D). In two independent clones of GOPC-deficient cells, protein abundance of LRRC8A and LRRC8C was unchanged (Figure 3D), showing that lack of GOPC did not result in loss of VRAC subunits. To test if UL56 targets VRAC subunits for proteasomal degradation, we treated cells with the proteasome inhibitors lactacystin and MG132. Proteasome inhibition has been reported to block HSV-1 infection ^31^. Therefore, instead of virus-infected cells, we used cells transduced with UL56 or GFP as a control. As expected, proteasome inhibitors increased the levels of ubiquitinated proteins (Figure 3E); in addition, GOPC levels were elevated in inhibitor-treated UL56-transduced cells. LRRC8A protein levels were reduced in UL56 expressing cells and this effect was partially rescued upon treatment with proteasome inhibitors (Figure 3E). Together, these data suggest that UL56 recruits one or multiple E3 ubiquitin ligases to LRRC8A and LRRC8C and thereby facilitates their ubiquitination and subsequent proteasomal degradation.

### UL56 inhibits VRAC-dependent cGAMP uptake and signalling

**Figure 4:**
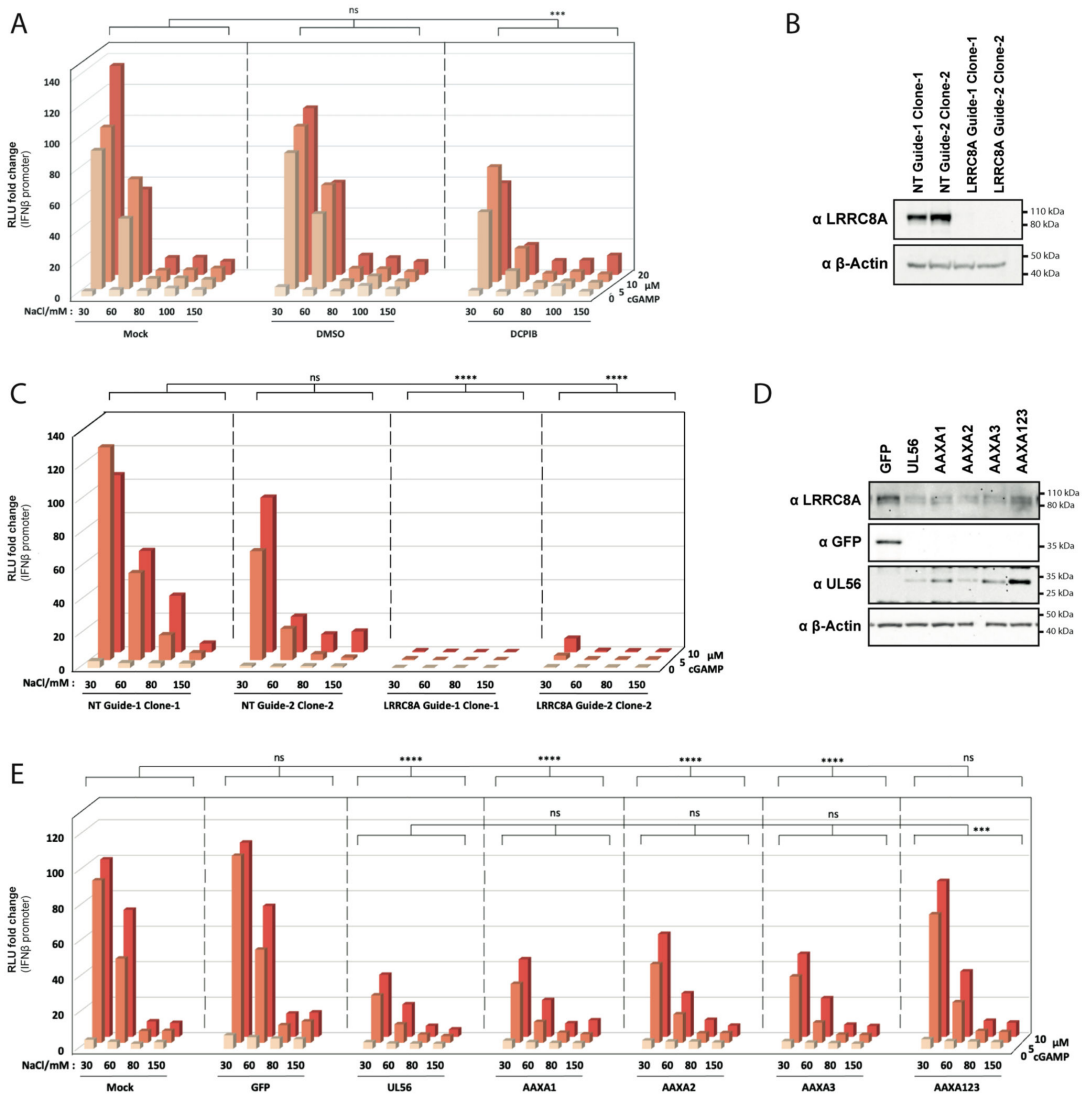
UL56 inhibits VRAC-mediated cGAMP uptake. (A) HEK293T cells were transiently transfected with three plasmids: p125-FLuc (*IFNβ* promoter reporter), pRL-TK (constitutively expresses Renilla luciferase) and pcDNA3.2-STING. After 24 hours, cells were incubated in low salt (NaCl) buffers containing cGAMP at the indicated concentrations. Where indicated, cells were also treated with vehicle control (DMSO) or DCPIB (20 μM). After one hour, these buffers were removed and replaced with medium. After an additional 24 hours, FLuc activity was measured and normalised to Renilla luciferase activity. The averages of all 150 mM NaCl / 0 μM cGAMP conditions was then set to 1. (B) HEK293 LRRC8A knockout clonal cell lines were generated using CRISPR-Cas9 and lysates were analysed by western blot using the indicated antibodies. (C) The cell lines shown in (B) were treated and analysed as in (A). (D) HEK293T stably transduced with GFP, UL56 or the indicated UL56 mutants were lysed and analysed by western blot using the indicated antibodies. (E) The cells shown in (D) were treated and analysed as in (A). Data are representative of three independent biological repeats. In (A), (C) and (E), averages of three technical replicates from one experiment are shown, and statistical analysis was done using grouped two-way ANOVA – Tukey. *=p<0.05, **=p<0.01, ***=p<0.001, ****=p<0.0001, ns=not significant. See also Figure S7.

To test the functional implications of our findings on cGAMP uptake and signalling, we developed a reporter cell system in which low salt concentrations trigger VRAC opening. We transiently transfected HEK293T cells, which are naturally STING-deficient ^32^, with a mix of three plasmids: (1) an expression plasmid for STING, (2) an *IFNβ* promoter-driven firefly luciferase reporter (p125-FLuc) and (3) a constitutively expressed Renilla luciferase as a transfection control (pRL-TK). Cells were then incubated with increasing doses of cGAMP added to the culture media, which contained decreasing salt concentrations. cGAMP at doses up to 20 μM did not trigger induction of the *IFNβ* promoter reporter when the NaCl concentration was 80 mM or higher (Figure 4A). However, at 60 mM NaCl, and more potently at 30 mM NaCl, cGAMP induced dose-dependent reporter expression (Figure 4A). This was consistent with the known osmolarity thresholds for VRAC opening ^19^ and the response was partially blocked by the VRAC inhibitor DCPIB (Figure 4A). To further validate that the response in this setting was VRAC-dependent, we generated LRRC8A knock-out HEK293 cells using CRISPR-Cas9 (Figure 4B). Two independently generated, LRRC8A-deficient clonal cell lines did not respond to cGAMP in medium containing low salt concentration (Figure 4C). Together, these data demonstrate that our reporter cell assay measures VRAC-dependent cGAMP uptake and signalling.

Next, we tested whether UL56 interferes with the response to extracellular cGAMP in this setting. HEK293T cells were lentivirally transduced to express GFP as control, wild-type UL56 or UL56 mutants in which the PPXY motifs were disabled individually or in combination. Expression of wild-type UL56 reduced the levels of LRRC8A (Figure 4D) and blocked IFNβ reporter induction compared to control cells expressing GFP (Figure 4E). These effects were also seen in cells expressing UL56 variants with a single mutated PPXY motif. However, upon mutation of all three PPXY motifs (construct AAXA123), UL56’s abilities to downregulate LRRC8A levels and to block cGAMP uptake and signalling were partially lost (Figure 4D-E). It is noteworthy that the levels of UL56-AAXA1, -AAXA3 and -AAXA123 proteins were increased compared to wild-type UL56, whilst the corresponding mRNAs were expressed at similar levels (Figures 4D and S7). UL56 does not contain lysine residues and hence cannot be ubiquitinated itself. We therefore speculate that tight binding of UL56 to proteins subsequently ubiquitinated by recruited E3 ligases may indirectly route UL56 to the proteasome. This may also explain elevated UL56-levels after proteasome inhibition (Figure 3E).

In sum, our data show that the HSV-1 protein UL56 antagonises VRAC subunits, including by targeting them for degradation, and that UL56 thereby interferes with uptake of cGAMP from the extracellular environment under conditions where VRAC is open.

### UL56 inhibits SLC46A2- and P2X7-mediated cGAMP uptake

**Figure 5:**
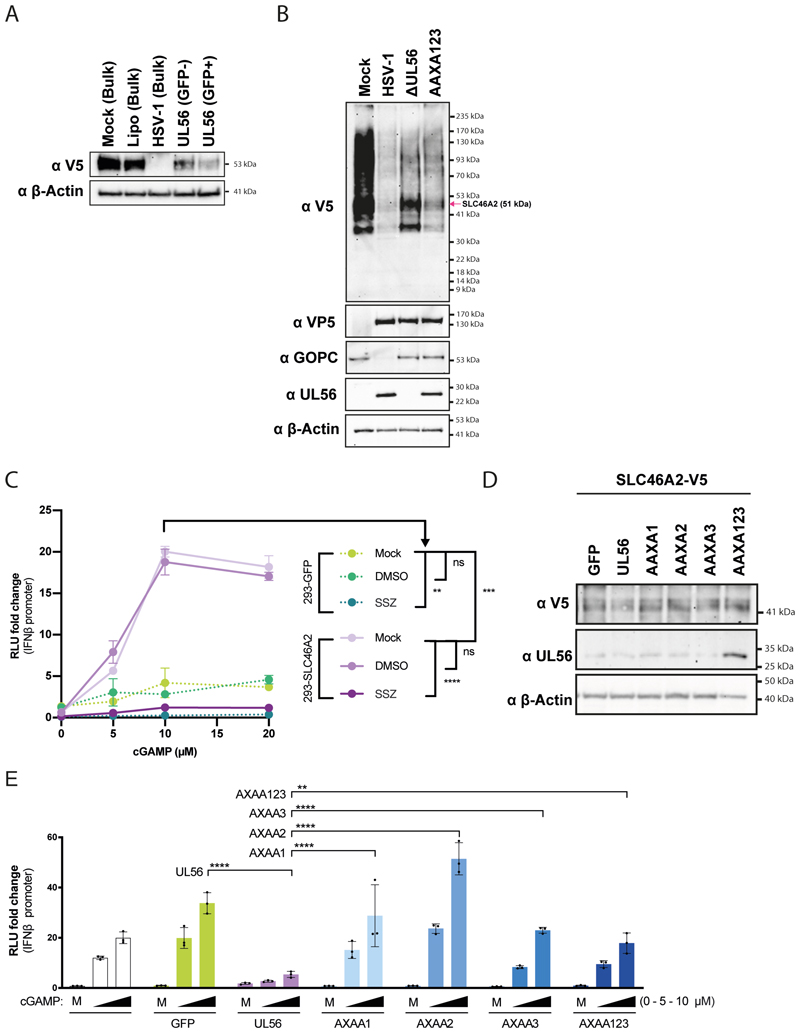
UL56 inhibits SLC46A2 mediated cGAMP uptake. (A) HEK293T cells stably transduced with SLC46A2-V5 were treated and analysed as described in Figure 3A. (B) HEK293T-SLC46A2-V5 were infected and analysed as described in Figure 3B. (C) HEK293T-SLC46A2-V5 cells were transfected as described in Figure 4A. After 24 hours, cGAMP was added to the medium at the indicated concentrations. Cells were also treated with vehicle control (DMSO) or SSZ (1 mM). After an additional 24 hours, *IFNβ* promoter reporter induction was assessed as in Figure 4A, setting the averages of all mock conditions to 1. (D) HEK293T-SLC46A2-V5 cells were additionally transduced with GFP, UL56 or the indicated UL56 mutants. Cell lysates were analysed by western blot using the indicated antibodies. (E) The cells shown in (D) were treated and analysed as in (C). Data in (A) and (D) are representative of two independent biological repeats. Data in (B), (C) and (E) are representative of three independent biological repeats. In (C) and (E), averages of three technical replicates from one experiment are shown with SD, and statistical analysis was done using grouped two-way ANOVA – Tukey. *=p<0.05, **=p<0.01, ***=p<0.001, ****=p<0.0001, ns=not significant. See also Figures S6 and S7.

**Figure 6:**
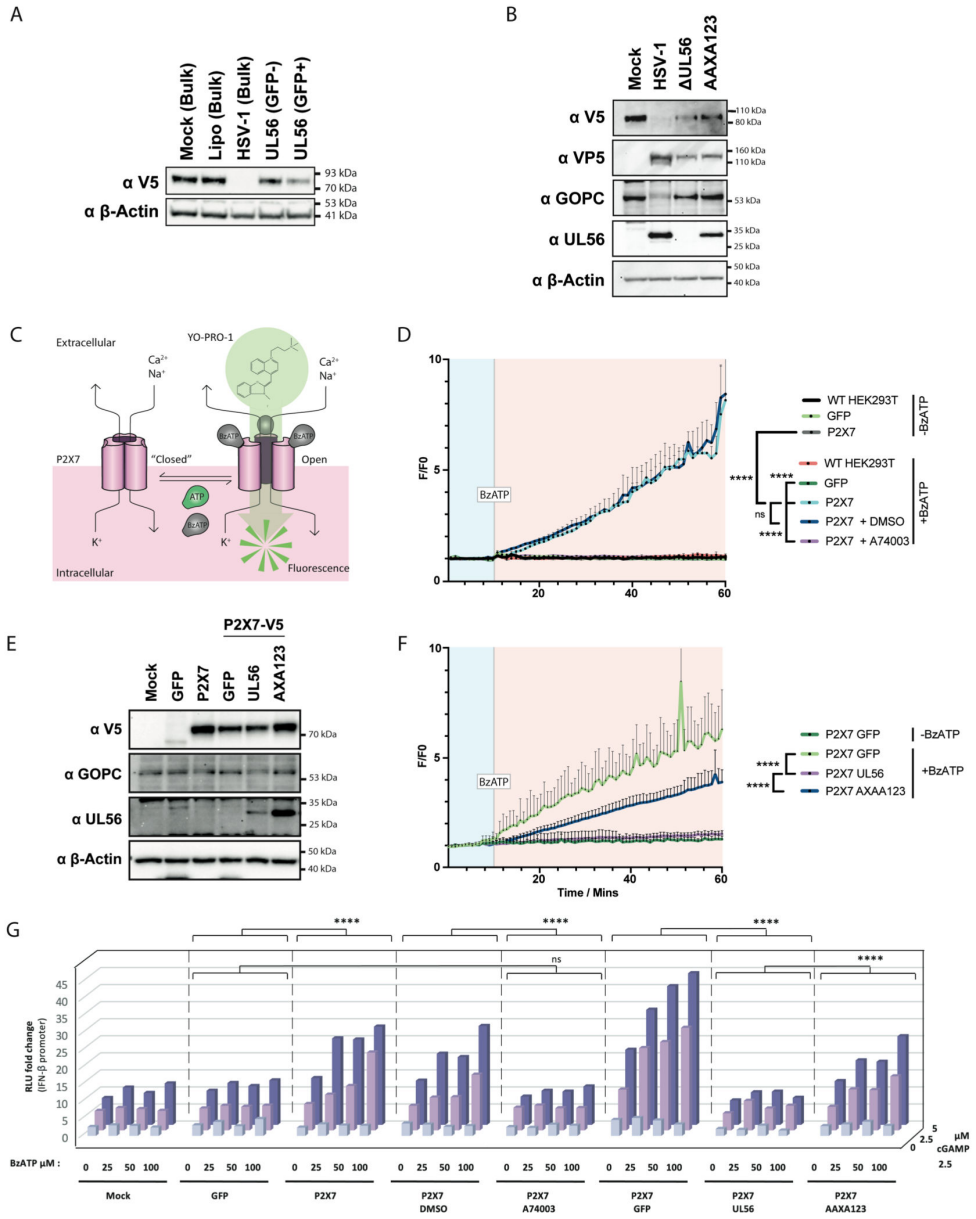
UL56 inhibits P2X7’s activity. (A) HEK293T cells stably transduced with P2X7-V5 were treated and analysed as described in Figure 3A. (B) HEK293T cells stably transduced with P2X7-V5 were infected and analysed as described in Figure 3B. (C) Schematic showing YO-PRO-1 uptake through P2X7. (D) HEK293T cells stably expressing GFP or P2X7-V5 were incubated YO-PRO-1 and green fluorescence was measured at 45 second intervals. After 10 minutes (vertical grey line), BzATP (80 µM) was added or not. P2X7-V5 cells were also treated with A74003 (100 mM) or DMSO as indicated. Fluorescence signals were normalised to the average fluorescence before BzATP was added (F/F0). (E) HEK293T-P2X7-V5 cells were additionally transduced with GFP, UL56 or the indicated UL56 mutants. Cell lysates were analysed by western blot using the indicated antibodies. (F) The cells shown in (E) were treated and analysed as in (D). (G) HEK293T-P2X7-V5 cells were transfected as described in Figure 4A. After 24 hours, BzATP and cGAMP were added to the medium at the indicated concentrations. After an additional 24 hours, *IFNβ* promoter reporter induction was assessed as in Figure 4A. The averages of all 0 μM BzATP / 0 μM cGAMP conditions was set to 1. Data are representative of three independent biological repeats. In (D) and (F) data points show averages of 3 technical replicates with SD, and statistical analysis was done using grouped two-way ANOVA – Tukey. Statistical analysis in (D), (F) and (G) was done using grouped two-way ANOVA – Tukey. *=p<0.05, **=p<0.01, ***=p<0.001, ****=p<0.0001, ns=not significant. See also Figures S6 and S7.

HSV-1 infection not only led to diminished protein levels of VRAC subunits, but also of SLC46A2 and P2X7 (Figures 1B, S2D and S2E). We therefore investigated if UL56 also antagonises SLC46A2 and P2X7. Using HEK293 cells stably transduced with SLC46A2-V5 or P2X7-V5, we repeated the transient transfection of UL56-GFP and the FACS enrichment of GFP+ cells. As with LRRC8A, we found that abundance of SLC46A2-V5 and P2X7-V5 was reduced in UL56-GFP-expressing cells (Figures 5A and 6A). Moreover, both SLC46A2-V5 and P2X7-V5 were strongly depleted in HEK293 cells infected with wild-type HSV-1 and protein levels were partially rescued in cells infected with HSV-1 ΔUL56 or HSV-1 UL56-AAXA123 (Figures 5B and 6B). This suggests that HSV-1 targets SLC46A2 and P2X7 for degradation in a UL56- and ubiquitination-dependent manner.

To determine whether UL56 interferes with cGAMP uptake through SLC46A2, we transfected HEK293 cells stably transduced with SLC46A2-V5 with STING and the *IFNβ* promoter reporter system described in Figure 4. Cells transduced with GFP served as a control and did not respond to cGAMP added to the culture medium (Figure 5C). In contrast, cells expressing SLC46A2-V5 showed a dose-dependent increase in reporter expression in response to extracellular cGAMP (Figure 5C). This response was blunted by treatment of cells with sulfasalazine (SSZ), an inhibitor of SLC19A1 and SLC46A2 ^16^. These results show that the response in this setting was mediated by SLC46A2. Next, we lentivirally expressed GFP or UL56 in HEK293-SLC46A2-V5 cells and used our *IFNβ* promoter reporter system to monitor the response to extracellular cGAMP (Figures 5D and 5E). cGAMP induced reporter expression in GFP-expressing control cells, and this was largely prevented by wild-type UL56. In contrast, all UL56 PPXY motif mutants tested did not antagonise reporter induction, which correlated with elevated levels of SLC46A2-V5 in cells expressing UL56 mutants (Figure 5D and 5E).

We next undertook similar functional experiments for P2X7 that forms a pore, which opens upon ATP binding and allows diffusion of molecules up to 0.9 kDa along concentration gradients ^33^. YO-PRO-1 is a membrane-impermeant cation of 629 Da that fluoresces upon binding nucleic acids and can be used with the ATP analogue BzATP to monitor P2X7-dependent uptake ^34–36^ (Figure 6C). We incubated HEK293T cells stably expressing GFP or P2X7-V5 with YO-PRO-1 in the presence or absence of BzATP (Figure 6D). Parental HEK293T cells and GFP-expressing cells did not take YO-PRO-1 up. However, HEK293T-P2X7-V5 cells showed increased fluorescence over time following BzATP addition to the medium, and this response was blunted in the presence of A74003, a P2X7 inhibitor ^37^ (Figure 6D). These results demonstrate that this assay measures P2X7-dependent YO-PRO-1 uptake. Next, we lentivirally expressed UL56 and UL56 mutants in P2X7-V5 cells and found that wild-type UL56 fully abrogated YO-PRO-1 uptake, while UL56-AAXA123 had a partial effect (Figures 6E and 6F). Finally, we analysed whether P2X7 antagonism by UL56 inhibits cGAMP uptake and signalling. We used our *IFNβ* promoter reporter system in HEK293-P2X7-V5 cells and found that P2X7 expression allowed for a BzATP-triggered response to cGAMP (Figure 6G). This response was blunted when these cells were treated with A74003 or expressed UL56 (Figure 6G). When all three PPXY motifs were mutated, UL56 partially lost its ability to block P2X7-mediated cGAMP uptake.

Taken together, these data indicate that UL56 antagonises cGAMP uptake and signalling via SLC46A2 and P2X7. Given that intact PPXY-motifs were required, it is likely these effects are at least in part mediated by ubiquitin ligase recruitment and degradation.

## Discussion

Signalling pathways employed by cells to detect virus invasion are critical for successful host defence. In turn, most - if not all - viruses have evolved means of antagonising or evading innate immune detection and/or downstream effector mechanisms. The targeting of a given cellular pathway or protein by a viral inhibitor therefore indicates that this cellular factor is an important barrier to viral replication and/or spread. Based on this notion, the presence of viral inhibitors has been proposed as a defining feature of HIV restriction factors, cellular proteins that interfere with HIV’s life cycle ^38^. Here, we employed the same rationale and postulated that viruses encode antagonists of cGAMP channels, transporters and pores. Using viruses representing six virus families, we screened protein levels of all known cGAMP conduits after infection of cells and report multiple examples of virus-triggered changes in abundance of these transmembrane proteins (Figure 1B).

HSV-1 infection led to reduced levels of four proteins involved in cGAMP transfer between cells: LRRC8A, LRRC8C, P2X7 and SLC46A2. A combination of kinetic analysis, evolutionary comparison and proteomic and functional studies led us to identify UL56, an HSV-1 protein that was required and sufficient for the effects on all four cellular proteins. UL56 is an adaptor protein and recruits cellular NEDD4 family E3 ubiquitin ligases through PPXY motifs ^30,39,40^. One target is GOPC, which is efficiently degraded via the ubiquitin-proteasome pathway in a UL56-dependent manner in HSV-1-infected cells ^30^. GOPC is a cellular trafficking factor for transmembrane proteins such as CFTR and TLR2 ^30,41^. It was therefore possible that the reduction in protein levels of cGAMP conduits was an indirect consequence of GOPC loss in infected cells, perhaps due to degradation after mislocalisation. However, LRRC8A and LRRC8C levels were unchanged in *GOPC*^*-/-*^ cells, ruling out this possibility. Instead, we favour a model in which UL56 recruits one or multiple E3 ubiquitin ligases to LRRC8A, LRRC8C, P2X7 and SLC46A2, targeting them for subsequent degradation. This was supported by our observation that HSV-1 expressing UL56 with disabled PPXY motifs could not target LRRC8A and LRRC8C (Figure 3B) and was less efficient than wild-type HSV-1 towards SLC46A2 and P2X7 (Figures 5B and 6B). Proteasome inhibition partially rescued LRRC8A levels in UL56 expressing cells (Figure 3E). In addition, it is possible that lysosomal turnover triggered by UL56-dependent ubiquitination plays a role in degradation of these proteins ^42^. It would be interesting to identify the E3 ligase(s) mediating ubiquitination of cGAMP conduits; however, such efforts are likely to be complicated due to redundancy between NEDD4 family E3 ligases ^43^. Another interesting question for future research is whether the three PPXY motifs in UL56 are redundant or play specialised roles in recruitment of specific E3 ligases. Such specificity might explain why mutating single PPXY motifs did not affect UL56-induced degradation of LRRC8A whereas mutating single motifs was sufficient to prevent the targeting of SLC46A2 (Figures 4D and 5D). It is possible that additional mechanisms contribute to the reductions in protein levels of cGAMP channels, transporters and pores in infected cells. This includes VHS-mediated degradation of cellular mRNAs ^44^. Indeed, we observed reduced abundance of *LRRC8A* and *LRRC8C* mRNAs in cells infected at higher MOIs (Figure 2B).

UL56 is a membrane-anchored protein present in infected cells and virions ^45,46^. Like many other viral proteins, UL56 appears to be multifunctional. Indeed, a recent study shows that UL56 also inhibits cGAS ^47^. Zheng and colleagues demonstrate that UL56 binds cGAS and that cGAMP synthesis by cGAS is inhibited by UL56 in an *in vitro* assay involving recombinant proteins ^47^. This indicates that UL56 antagonises cGAS and cGAMP conduits by different mechanisms. Future studies would benefit from the identification of UL56 mutations that selectively impact these two functions. These findings also raise the question as to why HSV-1 has evolved to inhibit cGAS in multiple ways, involving not only UL56 but also VP22 ^48^, UL37 ^49^, ICP8 ^50^ and VHS ^51^, and to target downstream STING signalling by yet another set of viral proteins ^25^. We speculate that no single viral antagonist can completely block the cGAS-STING pathway, at least not at all stages of the viral life cycle and in all cell types infected *in vivo*. Co-evolution of host and pathogen has likely driven the emergence of multiple, partially redundant, and partially effective mechanisms of viral cGAS-STING inhibition.

In mice and mouse cells, VRAC channels containing LRRC8A and LRRC8E transport cGAMP and thereby promote host defense against HSV-1 ^18^. Interestingly, we found that LRRC8A was not degraded in mouse embryonic fibroblasts infected with HSV-1 (Figure S4B). Whilst we cannot rule out that mouse LRRC8A or another VRAC subunit may be targeted by HSV-1 in other cell types, our data are suggestive of species differences. This observation also precluded *in vivo* experimentation in this study and may indicate that mouse models could overestimate the role of VRAC in the human response to HSV-1. Interestingly, human and mouse LRRC8A are 99% identical at amino acid level. We therefore speculate that UL56 cannot recruit mouse NEDD4 family E3 ligases to LRRC8A.

Our functional experiments confirm and extend previous work showing that VRAC, SLC46A2 and P2X7 mediate uptake of cGAMP into cells ^15–19^. In particular, cGAMP entry into cells upon ATP-triggered opening of the P2X7 pore was previously demonstrated only in mouse cells ^17^; here, we showed that human P2X7 transports cGAMP, too. An important question for future work is why cGAMP is imported and exported by so many different channels, transporters and pores. It is possible that these proteins (i) have cell type- and tissue-specific functions, (ii) play different roles in development, (iii) control immune responses to specific pathogens and/or in other disease settings such as cancer and (iv) may in some instances allow cGAMP passage non-specifically, for example due to the size of the pore formed or chemical similarities of cGAMP with canonical substrates. Whilst these questions remain largely open for future research, our observation that HSV-1 and VACV antagonise multiple cGAMP conduits supports the notion that they play important roles in antiviral immunity.

Taken together, we show that multiple viruses modulate the expression of several cGAMP channels, transporters and pores. HSV-1 was particularly adept at antagonising cGAMP conduits. Our mechanistic experiments established that the HSV-1 UL56 protein targeted these cellular proteins for degradation and thereby diminished the responsiveness of cells to extracellular cGAMP. The function of cGAMP channels, transporters and pores in host-pathogen interactions is an interesting dimension in cGAS-STING signalling, and our data are a resource for future studies in this direction.

## Limitations of the study

We chose a late time point (48 hours) and low MOIs to screen different viruses for antagonism of cGAMP channels (Figure 1). This approach includes effects that may happen late during viral life cycles or upon viral spread. However, bystander effects of infections on cell death, transcription and translation rates, and RNA and protein stability at late timepoints are likely. Although we demonstrate a specific, HSV-1 UL56-mediated mechanism, we did not systematically determine the relative contributions of this specific mechanism and non-specific shutoff mechanisms on downregulation of cGAMP conduits in infected cells. Our data show that UL56’s PPXY motifs are required for the targeting of cGAMP conduits, implicating one or multiple NEDD4 E3 ligases. This requires further experimental validation and testing to identify the ligase(s) involved, followed by analysis of biochemical interactions. The impact of viral antagonism of cGAMP conduits on virus replication and *in vivo* immune responses and pathogenesis remains an open question.

## Star Methods

### Resource Availability

#### Lead contact

Further information and requests for resources and reagents should be directed to and will be fulfilled by the lead contact, Jan Rehwinkel (jan.rehwinkel@imm.ox.ac.uk).

#### Materials availability

All unique reagents generated in this study are available from the Lead Contact with a completed Materials Transfer Agreement.

### Experimental Model And Study Participants Details

#### Cells

Cells were maintained in a humidified incubator at 37°C at atmospheric oxygen levels and 5% CO_2_. Adherent cells were passaged using Trypsin-EDTA (Thermo Fischer Scientific, 25200056). HEK293 and HEK293T cells (gifts from Caetano Reis e Sousa, The Francis Crick Institute, UK), HFFs (gift from Michael Weekes, University of Cambridge, UK) and HaCaT cells (gift from Leonie Unterholzner, University of Lancaster, UK) were cultured in Dulbecco’s modified Eagle medium (Thermo Fischer Scientific, 41965-039) supplemented with 10% (v/v) foetal calf serum (Sigma-Aldrich) and 2mM L-glutamine (Gibco, 25030081). MeWo cells were a gift from Graham Ogg (University of Oxford, UK) and were maintained in MEM supplemented with 10% (v/v) FCS, 2mM L-glutamine (Gibco), 1x Non-essential amino acids (Gibco), and 1mM sodium pyruvate (Gibco). Immortalised mouse embryonic fibroblasts were described previously ^53^.

#### Viruses

HSV-1 (KOS strain) was from ATCC (VR-1493) and mutant viruses (ΔUL56, AXAA-123) were described before ^30^. HSV-2 (strain 333) was described before ^54^. VZV ROka was a gift from Jeffrey Cohen (NIH, Bethesda, USA) and was used as described before ^55^. VACV (WR) was a kind gift from Michael Way (The Francis Crick Institute, UK). Human adenovirus type 5 engineered to express GFP and the Cre recombinase was from Vector Biolabs (1700). VSV-G pseudotyped HIV-1-GFP and HIV-2-GFP were made with pNL4-3-ΔE-EGFP ^56^ or HIV-2 ROD9 Δenv GFP ^57^ (kind gift from Nicholas Manel), respectively, and pVSV-G as described before ^8^. ZIKV (ZIKV/*H.sapiens*/Brazil/PE243/2015) was a kind gift from Alain Kohl (University of Glasgow, UK). IAV (PR8) was a kind gift from Paul Digard (University of Edinburgh, UK). IAV titres were determined using NP staining as previously described ^58^ and TCID50 doses converted to pfu/ml by multiplying by 0.7.

#### HSV-1 production and titration

To propagate HSV-1 stocks, Vero E6 cells were infected at an MOI of 0.003 in Minimum Essential Medium (MEM) and incubated until cells started to round up and detached from the flask (after three to four days). The supernatant was then spun at 21,000 rpm for two hours at 4°C in the OPTIMA XPN-80 ultracentrifuge in a SW 32 Ti swinging-bucket rotor. The pellet was resuspended in PBS. Virus stocks were titrated by overlaying Vero cells with serially diluted virus for 1.5 hours. The inoculum was then removed and carboxylmethyl cellulose (1.5% w/v) in DMEM (10% FCS, 2mM L-glutamine) was added. Cells were left for three days and then fixed with 3.7% v/v PFA in PBS before crystal violet staining (5% v/v in H_2_O). Upon desiccation, plaques were counted and stock concentration determined in pfu/ml.

### Method Details

#### Cloning

To create expression constructs, target genes were PCR amplified from cDNA libraries with Herculase II Fusion DNA Polymerase (Agilent Technologies, 600677-51). PCR reactions were resolved on 1% agarose gels and amplicons of correct size extracted using the QIAGEN gel extraction kit (QIAGEN, 28704). GoTaq (Promega, M780B) was used to add 5’ A overhangs and the resulting DNA product was inserted into the pCR8/GW/TOPO (Thermo Fisher Scientific, K250020) entry vector according to the manufacturer’s instructions. Genes were then shuttled into plenti6.3-V5 (BLAST), plenti6.3-V5 (PURO) or pCDNA3.2 using a Gateway L-R recombination kit (Invitrogen, 12538120). Constructed plasmids were propagated in and then extracted from bacterial cultures derived from single clones of transformed 5-alpha competent *E. coli* (New England Biolabs, C2987H) using a HiSpeed Plasmid Maxi Kit (QIAGEN, 12662). To create CRISPR constructs, the lentiCRISPRv2 plasmid was linearised by incubation with BsmBI-v2 (New England Biolabs, R07395) in r3.1 buffer (New England Biolabs, B60035) for one hour at 55°C. The digested product was then dephosphorylated by addition of alkaline phosphatase (ThermoFisher Scientific, EF0654) and incubation for one hour at 37°C. The resulting DNA species were resolved on 1% agarose gels and extracted using the QIAGEN gel extraction kit. Complimentary primers encoding sgRNA where purchased (Merck) with 5’-CACC overhangs on the sense and 5’-AAAC overhangs on the antisense strands. Guide sequences were as follows: NT Guide-1 5’-ACGGAGGCTAAGCGTCGCAA-3’, NT Guide-2 5’-CGCTTCCGCGGCCCGTTCAA-3’, LRRC8A Guide-1 5’-GGATCCTGAAGCCGTGGT-3’, LRRC8A Guide-2 5’-GGCACCAGTACAACTACG-3’, GOPC Guide-1 5’-GGAACATGGATACCCCGCCA-3’. After primers were annealed, they were phosphorylated with T4 PNK (New England Biolabs, M0201S). The overhangs created sticky ends enabling ligation with T4 ligase (New England Biolabs, B0202A) into the linearised lentiCRISPRv2 ^59^. All constructs were verified by Sanger sequencing (Source Bioscience).

#### Production of lentiviruses

Lentiviral particles were produced by transfection of HEK293T cells with either plenti6.3 or lentiCRIPSR-v2 constructs, pCMV-VSV-G and p8.91 using lipofectamine 2000 (Thermo Fisher Scientific, 11668030). Supernatants were collected 48 and 72 hours after transfection and filtered through a 0.45 μm Polyethersulfone (PES) membrane (Millex, SLHPO33RS). Cells were transduced in the presence of polybrene (8 μg/ml). Puromycin (1 µg/ml) or blasticidin (10 µg/ml) were used to select for stably transduced cells.

#### Generation of knockout cell lines

p125 HEK293 cells ^60^ were transduced with lentiviral vectors derived from lentiCRISPR-v2 constructs targeting *LRRC8A* or *GOPC*. Following antibiotic selection, clonal lines were obtained by limiting dilution. Clones were then screened by western blot.

#### Western blotting

50 μl of NP-40 lysis buffer (50 mM Tris, 150 mM NaCl, 2mM EDTA, 5% (v/v) glycerol, 1% (v/v) NP40, pH 7.4) supplemented with protease inhibitors (Cell Signalling - 5871S) was used to lyse 1×10^6^ cells. Insoluble cellular debris was pelleted, and supernatants were mixed with NuPage loading buffer (Invitrogen, NP0007) supplemented with 5% β-mercaptoethanol. Samples were heated for five minutes at 95°C. This step was omitted for SLC19A1, SLC46A2 and ABCC1. Lysates were then subjected to SDS-PAGE using 4-12% Bis-Tris precast gradient gels (Thermo Fisher Scientific, NP0321). Proteins were transferred to nitrocellulose membranes (BioRad, 1620112) using semi-dry transfer (BioRad, Trans-Blot Turbo) and blocked in TBS-N (10 mM Tris, 140 mM NaCl, 0.05% (v/v) NP-40, 5% (w/v) dried milk powder (Merck, 70166)), before probing with primary and HRP-coupled secondary antibodies. Signals were visualised using the chemiluminescent HRP substate ECL (Perkin and Elmer, NEL104001EA) and an iBright FL1000 instrument (Invitrogen). Band intensities were quantified using the densitometry analysis software available within the Thermo Fisher Connect Platform.

#### Immunoprecipitation and mass spectrometry analysis

HEK293T cells were stably transduced with lentiviruses derived from plenti6.3 LRRC8A-V5 (PURO) and plenti6.3 LRRC8C-V5 (PURO). Cells were infected with HSV-1 at an MOI of 10 for eight hours before lysis with NP-40 lysis buffer supplemented with protease inhibitors. Insoluble cellular debris was pelleted. Dynabeads (Invitrogen, 14311D) were coupled to 2.5 μg of α-V5 antibody (Biolegend, 680602) and added to each reaction. Input, unbound and IP samples were collected and analysed by western blot. Silver staining was performed using the Pierce Silver Stain kit (Thermo Fisher Scientific, 24600). LRRC8C interacting proteins were eluted specifically from the beads using an excess of V5 peptide. Eluted fractions were then digested with trypsin using the S-trap micro spin columns following the manufacturer’s protocol (PRofiti). In brief, SDS was added to the sample to 2 % final concentration. Samples were then reduced with 20 mM DTT for 30 minutes and alkylated with 40 mM iodoacetamide for a further 30 minutes at room temperature and in the dark. After that, samples were acidified with phosphoric acid (1.2 % final concentration) and proteins were precipitated with 90 % methanol / 100 mM TEAB (1/7 ratio sample/buffer). Samples were then loaded into the S-trap cartridge, washed three times with 90 % methanol / 100 mM TEAB buffer before adding 1.4 μg of trypsin (Promega). Trypsin digestion was carried out overnight at 37°C. Peptides were then sequentially eluted with 50 mM TEAB, 2 % formic acid and 2 % formic acid in 50 % acetonitrile solution, dried down using a centrifugal evaporator and resuspended in LC-MS/MS water containing 2 % acetonitrile, 0.1 % TFA.

20 % of tryptic peptides were analysed by liquid chromatography tandem mass spectrometry (LC-MS/MS) using Ultimate 3000 UHPLC (ThermoFisher Scientific) connected to an Orbitrap Fusion Lumos Tribrid (ThermoFisher Scientific). Peptides were separated using a 60 minute linear gradient from 2 % to 35 % buffer B (A: 5 % DMSO, 0.1 % formic acid; B: 5 % DMSO, 0.1% formic acid in acetonitrile) at 250 nl/min flow rate and analysed on the Orbitrap Fusion Lumos Tribrid (instrument control software v3.3). Data were acquired in data-dependent mode (DDA), with the advance peak detection (APD) enabled. Survey scans were acquired in the Orbitrap at 120 k resolution over a m/z range of 400 -1500, AGC target of 4e5 and S-lens RF of 30. Fragment ion spectra (MS/MS) were obtained in the Ion trap (rapid scan mode) with a Quad isolation window of 1.6, 40 % AGC target and a maximum injection time of 35 ms, with HCD activation and 28 % collision energy.

Data were analysed combining PEAKS-X+ software (Bioinformatics Solutions Inc.) for protein identification and ProgenesisQI (v4.1, non-linear dynamics, Waters) for label free quantitation. Briefly, data were searched against Human UniProt Swissprot database (Alignment ID -20200911_seq23155) and search parameters were set to: 10 ppm peptide and 0.5 Da MS/MS mass tolerance, respectively; trypsin; 2 missed cleavages; carbamidomethylation (C) as fixed modification; and oxidation (M), deamidation (NQ) and phosphorylation (STY) as variable. PEAKS-PTM identification outputs were exported after applying a 1 % FDR at PSM level and imported to Progenesis to generate relative abundances. The mass spectrometry data have been deposited to the ProteomeXchange Consortium via the PRIDE ^52^ partner repository with the dataset identifier PXD043229 (Reviewer account details: Username: reviewer_pxd043229@ebi.ac.uk; Password: 43G5stHF).

#### RT-qPCR

RNA was extracted using the RNeasy Plus Mini Kit (Qiagen, 74136) and annealed to random hexamers (QIAGEN, 79236) to prime production of cDNA by SuperScript II reverse transcriptase (Thermo Fisher Scientific, 18064014) following the manufacturer’s instructions. cDNA was diluted 1:5 in water, and then 2 μl were used as the template for real-time PCR using either the Taqman universal PCR master mix (Applied Biosystems, 4304437) or EXPRESS SYBR™ GreenER™ qPCR Supermix (Thermo Fisher Scientific, 11784200) on the Quant Studio 7 flex real-time PCR machine (Applied Biosystems). *GAPDH* was used as the housekeeping gene for normalisation using the *delta-delta Ct* method described by Livak and Schmittgen ^61^. TaqMan probes for *GAPDH* (Hs02758991_g1), *LRRC8A* (Hs01555916_m1) and *LRRC8C* (Hs00943621_m1) were from Invitrogen. The primers used to quantify expression of *GAPDH* (5’-CATGGCCTTCCGTGTTCCTA-3’ and 5’-CCTGCTTCACCACCTTCTTGAT-3’) and *UL56* (5’-ACCAGCGACGAACGCAAAAC-3’ and 5’-ACCACCCCAAATACAGCATGGC-3’) were from Merck.

#### Dual luciferase assay

4×10^4^ HEK293T cells were seeded into 96 well plates. 24 hours later, cells were transfected with p125-FLuc ^62^ (20 ng per well), pRL-TK (5 ng) and pcDNA3.2-STING (10 ng) using lipofectamine 2000 (0.4 μl). 48 hours after seeding, cells were treated with 2’3’-cGAMP (Stratech, B8362-APE). For LRRC8A:C activation, 2’3’-cGAMP was applied in either an isotonic (150 mM NaCl, 6 mM KCl, 1 mM MgCl_2_, 1.5 mM CaCl_2_, 10 mM glucose, 10 mM HEPES, pH 7.4) or hypotonic buffer with reduced NaCl concentration for one hour. For P2X7 activation, 2’3’-cGAMP was applied with bzATP (Biotechne, 3312) in DMEM. As indicated in the Figures, inhibitors of 2’3’-cGAMP transporters, channels or pores were applied, including DCPIB (Cayman, 34064), A73004 (Merck, 5083170001) and sulfasalazine (Cayman, 15025). Luminesce was then read using the dual luciferase assay kit (Promega, E1960). Firefly luciferase levels (indicative of *IFN*β promoter activation) were divided by Renilla luciferase levels to normalise for transfection efficiency.

#### Fluorescence activated cell sorting (FACS)

HEK293T cells were transfected with either pEXP103 GFP-UL6 or pEXP103 GFP-UL56 ^63^ with lipofectamine 2000. After 24 hours, cells were trypsinised and stained with violet live dead viability dye (Thermo Fischer Scientific, L34955) in PBS. Cells were then sorted by a trained operator on the BD FACS Aria Fusion Flow Cytometer into GFP^+^ and GFP^-^ populations. A small proportion of the sorted cells were re-run through the FACS Aria to check the purity of the sorted populations. Directly after sorting, cells were lysed for western blot as described. Data were analysed using the software package FlowJo (v10.8).

#### YOPRO-1 uptake assay

This assay was based upon a previously published protocol ^36^. In short, 1×10^5^ HEK293 cells were seeded into flat bottom 96 well plates pre-treated with 10% (v/v) collagen (Sigma, C8919) in PBS for 20 minutes before use. Assay buffer (2 mM KCl, 0.1 mM CaCl_2_, 13 mM Glucose, 147 mM NaCl, 10 mM HEPES, pH 7.3) containing YO-PRO1 dye (Life Technologies, Y3603) was added to the cells. Then cells were excited with a laser (485-15 nm) and emission (528-20 nm) was measured from the bottom of the plate using the CLARIOstar plate reader’s (BMG Lab Tech) orbital averaging function. After the first 10 minutes, bzATP was added and fluorescence readings were taken every 45 seconds.

### Quantification And Statistical Analysis

All experiments were performed three times or more independently under similar conditions, unless specified otherwise in figure legends. Statistical significance was calculated as described in the figure legends; p < 0.05 was considered significant. GraphPad Prism 10 software was used to generate graphs and to perform statistical analysis.

## Supplementary Material

Combined Supplementary Materials

Key Resources Table

Table S2

## Data Availability

Data are available in the manuscript and its associated supplementary files. The mass spectrometry proteomics data have been deposited to the ProteomeXchange Consortium via the PRIDE ^52^ partner repository with the dataset identifier PXD043229. This paper does not report original code. Any additional information required to reanalyze the data reported in this paper is available from the lead contact upon request.
