# Supplementary material for: HSV-1 employs UL56 to antagonise expression and function of cGAMP channels
Source: Cell Rep. Author manuscript; Available in PMC 2025 Jul 29. (PMC7617956; doi:10.1016/j.celrep.2024.114122)
Supplement: Combined Supplementary Materials [file EMS206900-supplement-Combined_Supplementary_Materials.pdf]

Supplementary figures and legends

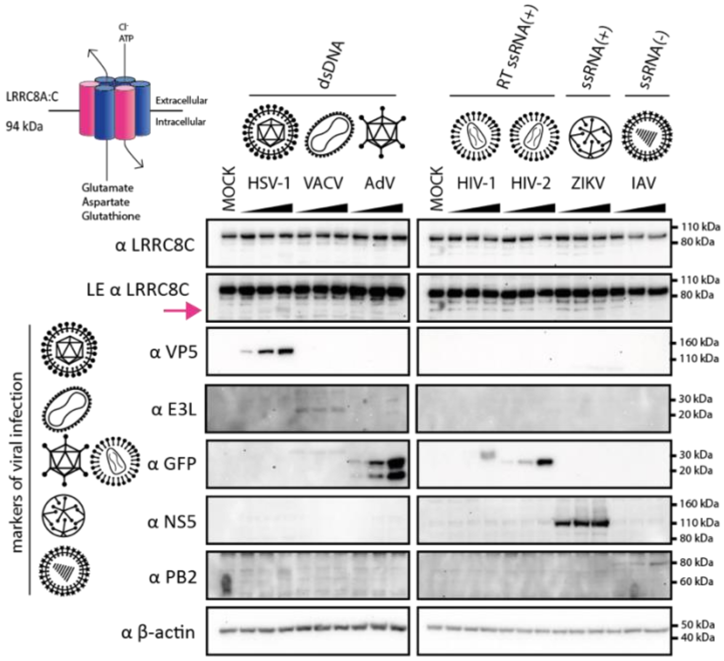

Figure S1. LRRC8C abundance during viral infection. Related to Figure 1

HEK293 cells were infected and analysed as in Figure 1A. LE, long exposure. The arrow indicates a faster migrating band detected by the α-LRRC8C antibody.

Data are representative of two (AdV) and three biological repeats (all other viruses).

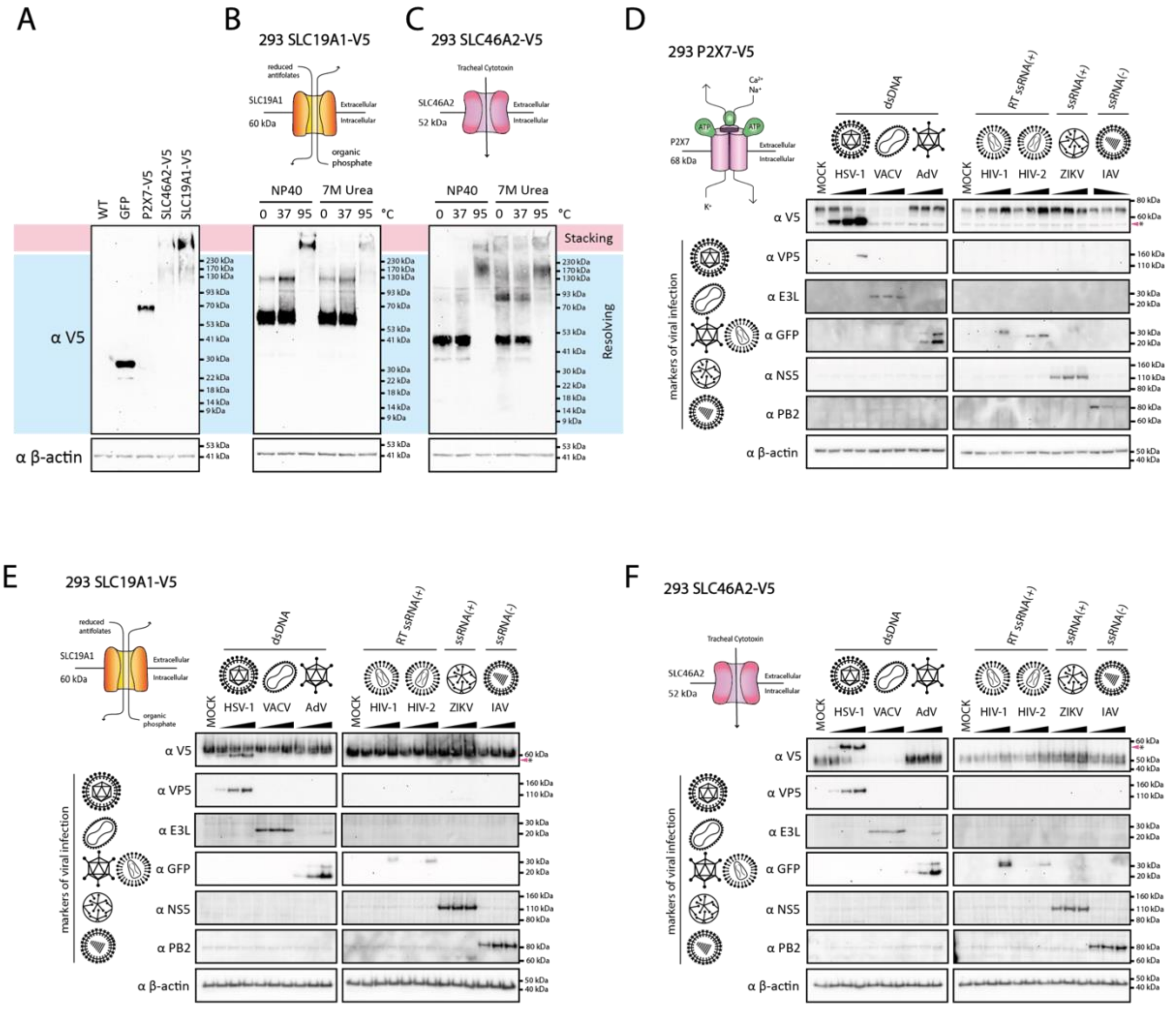

Figure S2. P2X7, SLC46A2 and SLC19A1 abundance during viral infection. Related to Figure 1

(A) Western blot analysis using the indicated antibodies and lysates from parental HEK293 cells (WT) and cells stably transduced with GFP-V5, P2X7-V5, SLC46A2-V5 or SLC19A1-V5. Pink and blue boxes indicate the stacking and resolving portions of the gel. β-Actin served as a loading control.

(B, C) SLC19A1-V5 or SLC46A2-V5 stably transduced cells were lysed in NP40 lysis buffer in the presence or absence of 7M urea, treated for five minutes at the indicated temperatures and analysed as in (A).

(D-F) P2X7-V5 (D), SLC19A1-V5 (E) and SLC46A2-V5 (F) transduced cells were infected and analysed as described in Figure 1A. Asterisks indicate a cross-reactivity of the α-V5 antibody with an HSV-1 protein.

Data in (A-D) are representative of three biological repeats. The experiments shown in (E) and (F) were performed once.

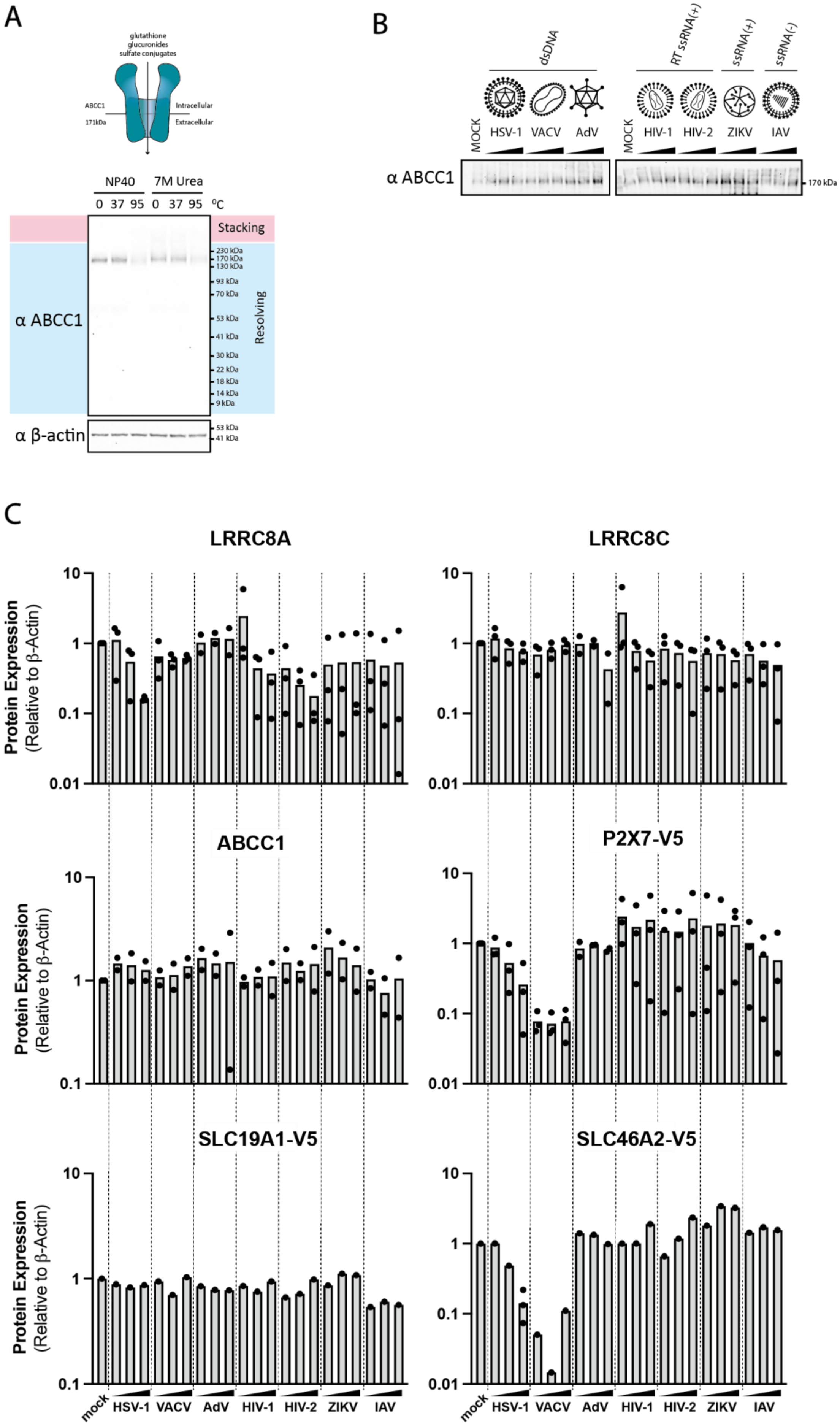
A
glutathione
glucuronides
sulfate conjugates
ABCC1
171kDa
Intracellular
Extracellular
NP40
7M Urea
0 37 95 0 37 95 °C
Stacking
α ABCC1
Resolving
230 kDa
170 kDa
130 kDa
93 kDa
70 kDa
53 kDa
41 kDa
30 kDa
22 kDa
18 kDa
14 kDa
9 kDa
α β-actin
53 kDa
41 kDa
B
dsDNA
RT ssRNA(+)
ssRNA(+)
ssRNA(-)
MOCK
HSV-1
VACV
AdV
MOCK
HIV-1
HIV-2
ZIKV
IAV
α ABCC1
170 kDa
C
LRRC8A
LRRC8C
ABCC1
P2X7-V5
SLC19A1-V5
SLC46A2-V5
Protein Expression
(Relative to β-Actin)
10
1
0.1
0.01
mock
HSV-1
VACV
AdV
HIV-1
HIV-2
ZIKV
IAV

Figure S3. ABCC1 abundance during viral infection. Related to Figure 1

(A) HEK293 cells were lysed in NP40 lysis buffer in the presence or absence of 7M urea. Lysates were incubated for five minutes at the indicated temperatures and then analysed by immunoblot as in Figure S2B.

(B) HEK293 cells stable expressing SLC46A2-V5 were infected and analysed as in Figure 1A. Please note that membranes used in Figure S2F were re-probed with an α-ABCC1 antibody. Please see Figure S2F for viral protein and loading controls.

(C) Levels of the indicated proteins were quantified by densitometry using the western blots shown in Figure 1A (LRRC8A), S1 (LRRC8C), S3B (ABCC1), S2D (P2X7-V5), S2E (SLC19A1-V5) and S2F (SLC46A2-V5). Corresponding repeat experiments were quantified in the same way. Data were normalised to the β-Actin signal, set to 1 for mock infected cells, and repeat data were pooled. Each point shows data from an independent experiment and bars show the average. Please see the legend to Figure 1A for further details.

Data in (A) and (B) are representative of three and two biological repeats, respectively.

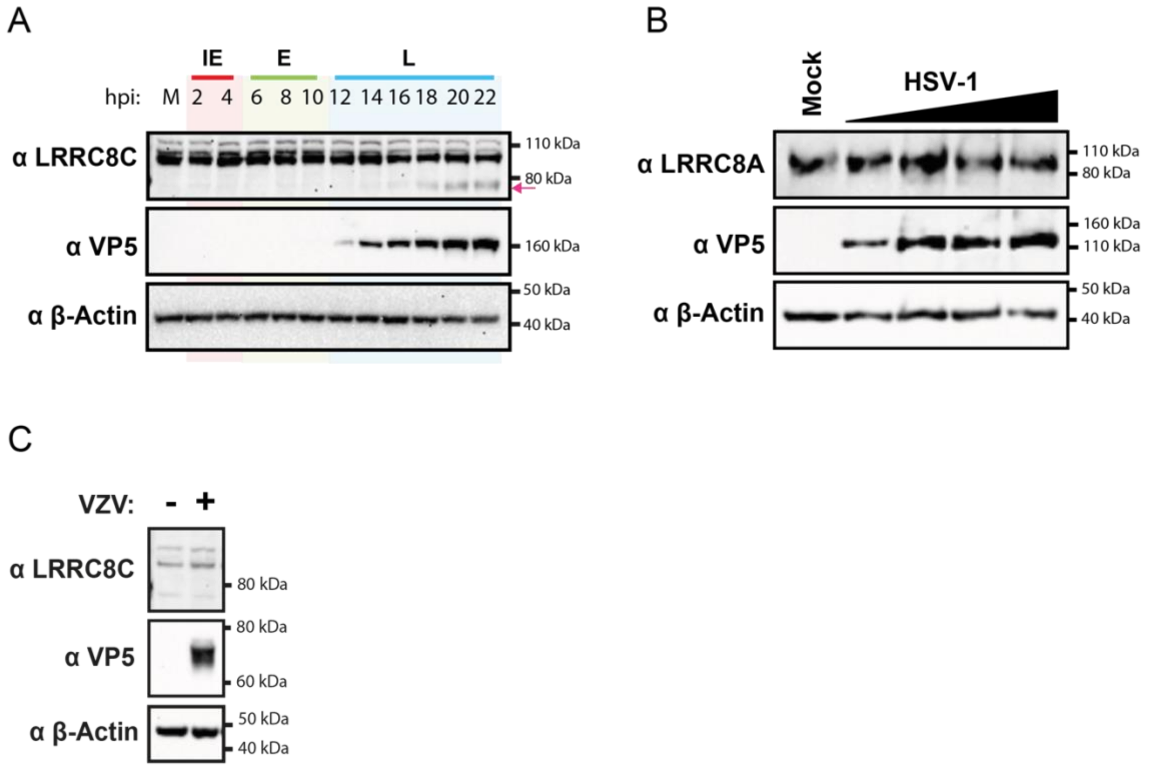

Figure S4. VRAC antagonism by herpesviruses. Related to Figure 2

(A) The experiment shown in Figure 2C was repeated using an α-LRRC8C antibody. The arrow indicates a faster migrating band detected by the α-LRRC8C antibody.

(B) Immortalised mouse embryonic fibroblasts were infected with HSV-1 (MOI = 1, 10, 30 and 100) for 24 hours. The indicated proteins were detected by western blot. β-Actin served as a loading control.

(C) The experiment shown in Figure 2G was repeated using an α-LRRC8C antibody.

Data in (A) are representative of two biological repeats. Data in (B) and (C) are representative of three biological repeats.

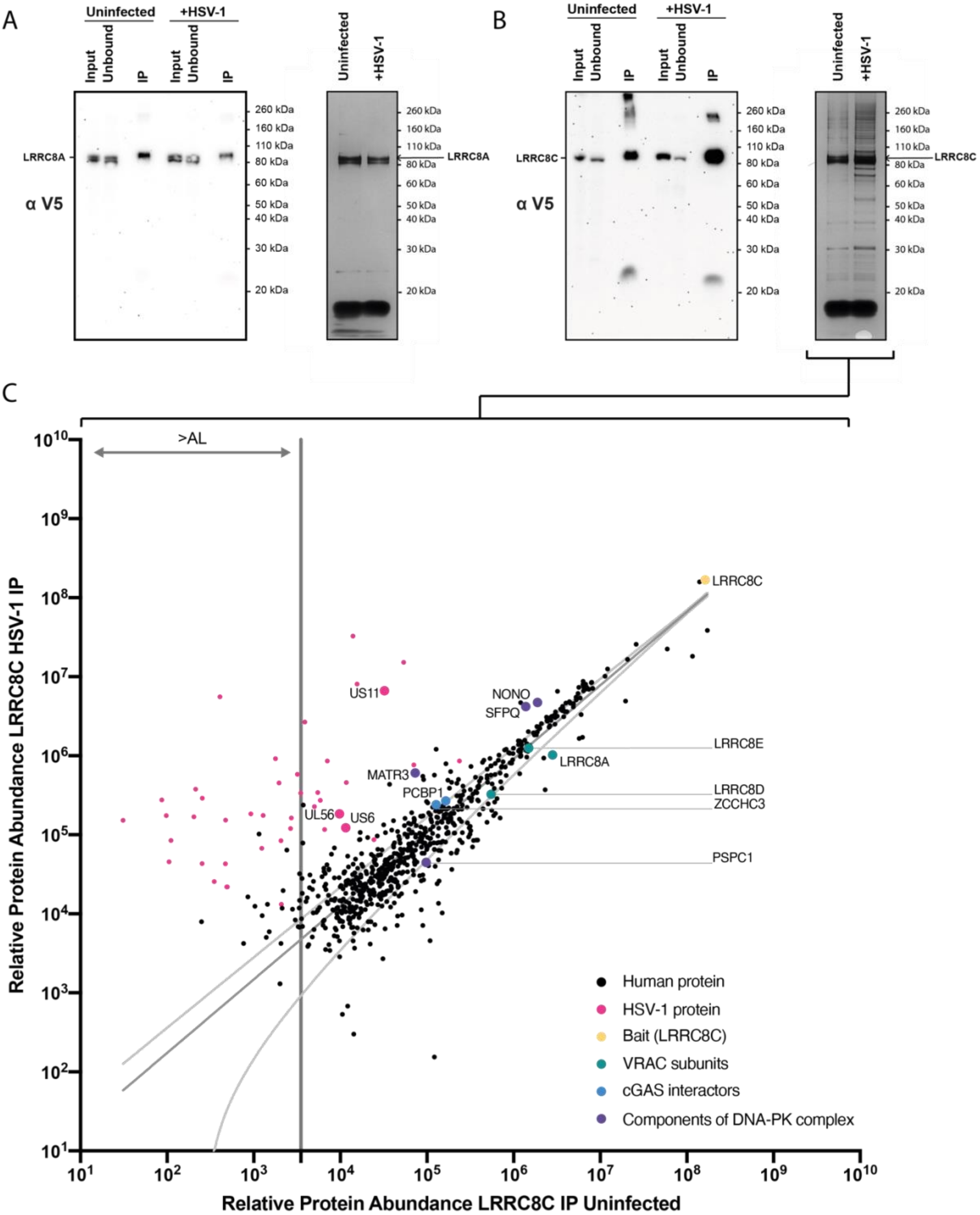

Figure S5. Immunoprecipitation of LRRC8A and LRRC8C from HSV-1 infected cells. Related to Figure 2

(A, B) HEK293 cells stably transduced with LRRC8A-V5 (A) or LRRC8C-V5 (B) were infected or not with HSV-1 (MOI = 10). Eight hours after infection, cells were lysed, and V5-tagged proteins were immunoprecipitated with an α-V5 antibody. Input, unbound and precipitated samples were analysed by immunoblot using the α-V5 antibody (left panels). Precipitated samples were also analysed by polyacrylamide gel electrophoresis and silver staining (right panels). Bait proteins are indicated.

(C) LRRC8C precipitates were analysed by mass spectrometry. PEAKS software was used to identify peptides and to align them to the Human UniProt Swissprot database (Alignment ID - 20200911_seq23155). The progenesis label free quantification software was used to assign relative values of protein abundance. The abundance limit (AL) was set to exclude lowest 3% of cellular proteins. Nonlinear regression shows average protein abundance

(dark grey line) with proteins above enriched and below depleted in LRRC8C-V5 immunoprecipitates from HSV-1-infected cells. Human proteins are shown in black and HSV-1 proteins in pink. LRRC8C and other VRAC subunits are highlighted in yellow and green, respectively. Proteins associated with cGAS or DNA-PK are shown in blue and purple, respectively. Candidate HSV-1 proteins are highlighted with large dots.

Data in (A) and (B) are representative of two biological repeats. Data in (C) are from one experiment and light grey lines show 95% confidence limits.

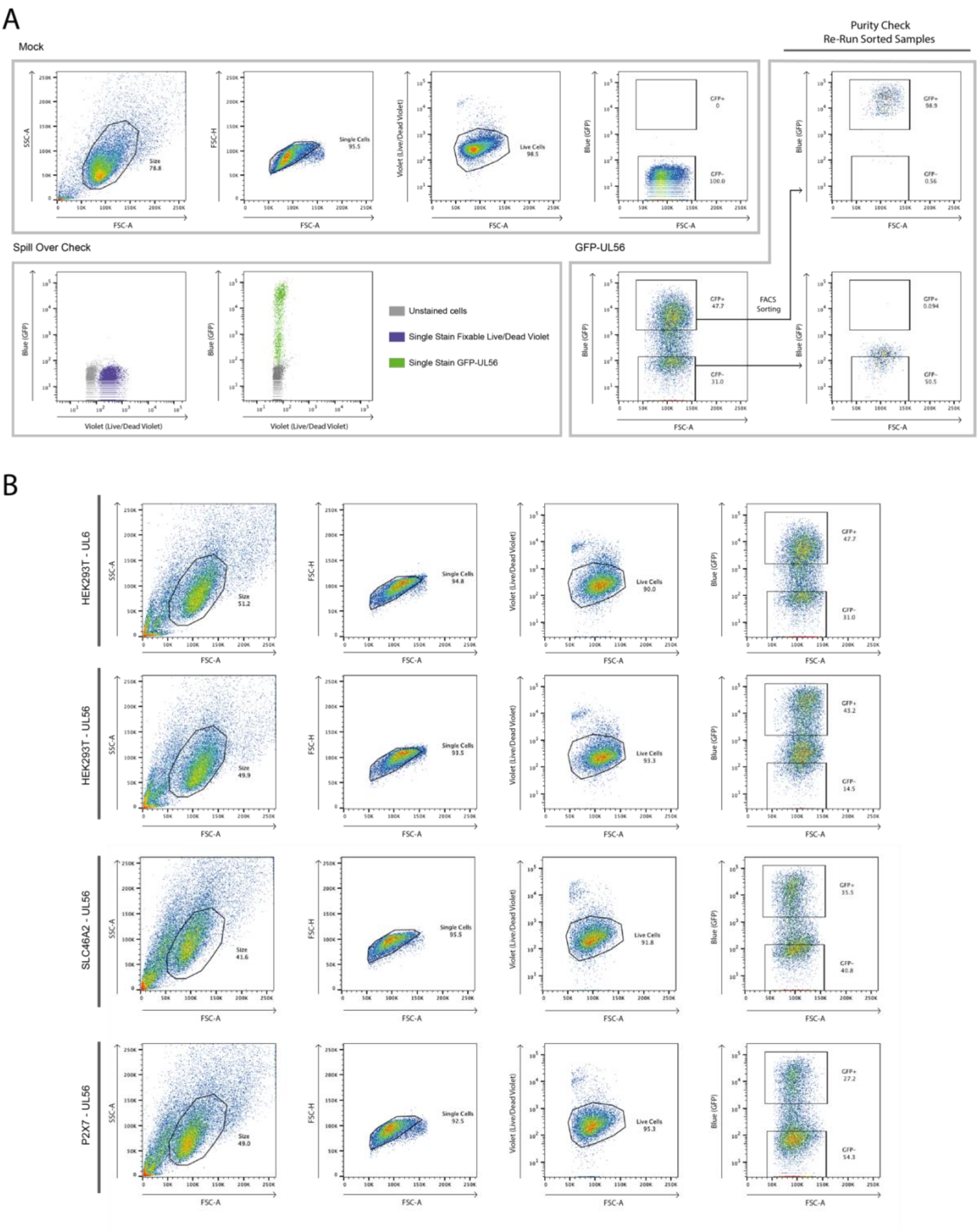

Figure S6. FACS gating strategy. Related to Figures 3, 5 and 6

(A) HEK293T cells were transfected as described in Figure 3A. Cells were stained with a fixable live/dead dye. The gating strategy is shown in the top left panel for mock transfected cells. The bottom left panel shows single stain controls. A small proportion of the sorted cells were purity checked by re-running the samples on a FACS analyser (right panel; UL56-GFP transfected cells).

(B) HEK293T cells transfected with UL6-GFP or UL56-GFP (rows 1 and 2) and HEK293 cells stably transduced with SLC46A2-V5 or P2X7-V5 and transfected with UL56-GFP (rows 3 and 4) were sorted into GFP- and GFP+ populations. The gating strategy is shown.

Data are representative of three biological repeats.

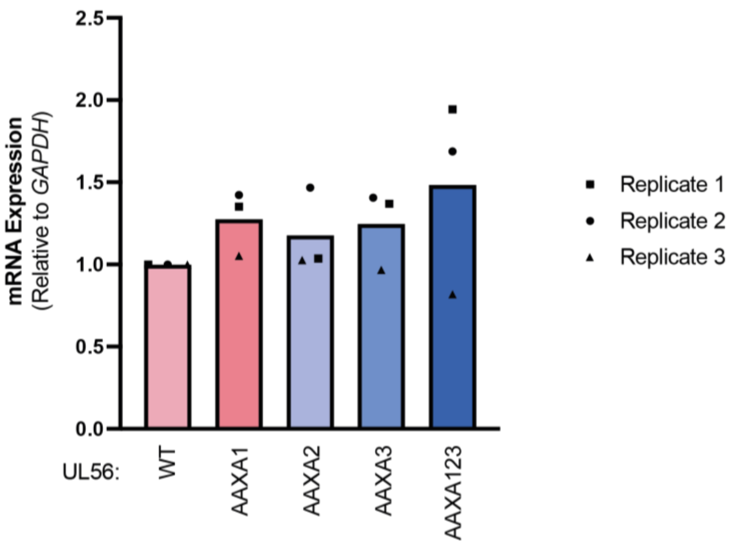

Figure S7. *UL56* mRNA expression. Related to Figures 4, 5 and 6
Total RNA was extracted from HEK293T stably transduced with GFP, UL56 or the indicated UL56 mutants. *UL56* mRNA levels were determined by RT-qPCR. Data were analysed by the comparative Ct method, normalised to *GAPDH* and set 1 for WT UL56.
Data are pooled from three independent biological repeats.

Supplementary tables

Table S1. Identification of an HSV-1 antagonist of LRRC8A and LRRC8C. Related to Figure 2H

HSV-1 genes and their homologs in HSV-2 and VZV are shown in columns 1-3 (based on [1,2]). Evidence and criteria for progressive exclusion (from left to right) as a factor targeting VRAC subunits are shown at the top. Excluded HSV-1 ORFs are highlighted by grey shading.

Evidence	HSV-2 degrades LRRC8A (Figure 2F).	VZV does not degade LRRC8A (Figure 2G).	LRRC8A is degraded late during viral infection (Figure 2C and D).	Gene detectable in LRRC8C IP from HSV-1 infected cells (Figure S5).	Gene detectable at a high abundance within LRRC8C IP from HSV-1 infected cells (Figure S5).
**Exclusion critieria**	HSV-1 genes with no homolog in HSV-2	HSV-1 genes with a homolog in VZV	Genes in immediate early (α) or early (β) kinetic class	Not detected in LRRC8C IP	Detected in LRRC8C IP at low abundance
**HSV-1 Gene Name and Aliases**	**HSV-2 Gene Name and Aliases**	**VZV Gene Name and Aliases**	**Kinetic Class**	**Detected in LRRC8C IP**	Detected in LRRC8C IP > Abundance Limit
UL56	UL56	No homolog	Leaky late γ1 - (Ushijima et al., 2008 & Koshhizuka et al., 2002)	yes	yes
US6, gD	US6, gD	No homolog	Leaky late γ1 - (Garvey et al., 2014 & Fox et al., 2017)	yes	yes
US11	US11	No homolog	True late γ2 - (Liu et al., 2019)	yes	yes
UL45, EV45	UL45	No homolog	True late γ2 - (Visalli & Brandt 2008)	yes	
US2	US2	No homolog	True late γ2 - (Fox et al., 2017)	yes	
γ134.5	γ134.5	No homolog	Leaky late γ1 - (Chou & Roizman 1986)		
UL9.5	Unclear if homolog exists	Unclear if homolog exists	True late γ2 - (Baradaran et al., 1994)		
UL27.5	UL27.5	No homolog	True late γ2 - (Chang et al., 1998)		
UL43.5	UL43.5	No homolog	Late γ - (Ward et al., 1996)		
US4, gG	US4, gG	No homolog	Leaky late γ1 - (Garvey et al., 2014)		
US5, gJ	US5, gJ	No homolog	Leaky late γ1 - (Aubert et al., 2008)		
US8.5	US8.5	No homolog	Leaky late γ1 - (Georgopoulou et al., 1995)		
UL12.5	UL12.5	No homolog	Early β - (Reuven et al., 2004 & Draper et al., 1986)		
US12, α47, ICP47	US12, α47, ICP47	No homolog	Immediate early α - (Chen et al., 2020)		
α0, ICP0 RL2	α0, ICP0	α0, ICP0 RL2			
α4, ICP4	α4, ICP4	α4, ICP4		yes	
US3	US3	US3		yes	yes
US1, α 22, ICP22	US1, α 22, ICP22	US1, α 22, ICP22		yes	
US1.5	US1.5	US1.5			
US7, gI	US7, gI	US7, gI		yes	yes
US8, gE	US8, gE	US8, gE		yes	
US9	US9	US9			
US10	US10	US10		yes	
UL1, gL	UL1, gL	UL1, gL		yes	
UL2	UL2	UL2			
UL3, NP03	UL3	UL3		yes	
UL4	UL4	UL4			
UL5	UL5	UL5			
UL6	UL6	UL6		yes	yes
UL7	UL7	UL7			
UL8	UL8	UL8			
UL8.5	UL8.5	UL8.5			
UL9, OBP	UL9	UL9		yes	
UL10	UL10	UL10			
UL11	UL11	UL11			
UL12, AN	UL12	UL12		yes	
UL13	UL13	UL13		yes	
UL14	UL14	UL14			
UL15	UL15	UL15			
UL15.5	UL15.5	UL15.5			
UL16	UL16	UL16			
UL17	UL17	UL17			
UL18, TRX2	UL18	UL18		yes	
UL19, MCP, VP5	UL19	UL19		yes	
UL20	UL20	UL20			
UL21	UL21	UL21			
UL22, gH	UL22	UL22		yes	yes
UL23, KITH, TK	UL23	UL23		yes	
UL24	UL24	UL24		yes	
UL25, CVC2	UL25	UL25		yes	
UL26, SCAF	UL26	UL26		yes	
UL26.5	UL26.5	UL26.5			
UL27 gB	UL27	UL27		yes	
UL28	UL28	UL28			
UL29	UL29	UL29			
UL30, DPOL	UL30	UL30		yes	
UL31 NEC1	UL31	UL31		yes	yes
UL32	UL32	UL32			
UL33	UL33	UL33			
UL34, NEC2	UL34	UL34		yes	
UL35	UL35	UL35			
UL36	UL36	UL36			
UL37	UL37	UL37		yes	
UL38	UL38	UL38		yes	
UL39, RIR1	UL39	UL39		yes	
UL40, RIR2	UL40	UL40		yes	
UL41 vhs, SHUT	UL41	UL41		yes	yes
UL42	UL42	UL42		yes	
UL43	UL43	UL43			
UL44, gC	UL44	UL44		yes	
UL46, TEG1	UL46	UL46		yes	yes
UL47 TEG5	UL47	UL47		yes	yes
UL48 vp16	UL48	UL48		yes	yes
UL49 vp22	UL49	UL49		yes	yes
UL49.5	UL49.5	UL49.5			
UL50, DUT	UL50	UL50		yes	yes
UL51	UL51	UL51			
UL52	UL52	UL52			
UL53	UL53	UL53			
UL54, α27	UL54, α27	UL54, α27		yes	yes
UL55, TEG6	UL55	UL55		yes	
ORF-O	No homolog	No homolog			
ORF-P	No homolog	No homolog			
UL20.5	No homolog	No homolog			

Supplementary references

1. Baines, J.D., and Pellett, P.E. (2007). Genetic comparison of human alphaherpesvirus genomes. In Human Herpesviruses: Biology, Therapy, and Immunoprophylaxis, A. Arvin, G. Campadelli-Fiume, E. Mocarski, P.S. Moore, B. Roizman, R. Whitley, and K. Yamanishi, eds.

2. Roizman, B., and Campadelli-Fiume, G. (2007). Alphaherpes viral genes and their functions. In Human Herpesviruses: Biology, Therapy, and Immunoprophylaxis, A. Arvin, G. Campadelli-Fiume, E. Mocarski, P.S. Moore, B. Roizman, R. Whitley, and K. Yamanishi, eds.
