# Supplementary material for: HSV-1 employs UL56 to antagonise expression and function of cGAMP channels
Source: Cell Rep. Author manuscript; Available in PMC 2025 Jul 29. (PMC7617956; doi:10.1016/j.celrep.2024.114122)
Supplement: Key Resources Table [file EMS206900-supplement-Key_Resources_Table.docx]

**KEY RESOURCES TABLE**

| REAGENT or RESOURCE | SOURCE | IDENTIFIER |  |
| --- | --- | --- | --- |
| Antibodies | | |  |
| LRRC8A | Santa Cruz Biotechnology | Cat# sc-517113; RRID: AB_2928142 |  |
| LRRC8C | 2B Scientific | Cat# MBS711316; RRID: AB_2864353 |  |
| ABCC1 | Abcam | Cat# ab260038; RRID: AB_2889834 |  |
| GOPC | Abcam | Cat# ab133472; RRID: AB_11156985 |  |
| Ubiquitin | Cell Signaling Technology | Cat# 3933S; RRID: AB_2180538 |  |
| b-Actin | Sigma Aldrich | Cat# A3854; RRID: AB_262011 |  |
| VP5 (HSV-1) | Santa Cruz Biotechnology | Cat# sc-56989; RRID: AB_629628 |  |
| UL56 (HSV-1; polyclonal) | Crump lab (ref. 30) | N/A |  |
| E3L (VACV) | gift from Vincenzo Cerundolo (University of Oxford, UK) | Clone TW2.3; RRID: AB_2892148 |  |
| GFP | Sigma Aldrich | Cat# 11814460001; RRID: AB_390913 |  |
| NS5 (ZIKV; polyclonal) | gift from Andres Merit (University of Tartu, Estonia) | N/A |  |
| PB2 (IAV) | GeneTex | Cat# GTX125925; RRID: AB_11170601 |  |
| ORF62 (VZV) | Meridian Life  Science | Cat# **C05107MA**; RRID: AB_1772162 |  |
| V5 (used in Figure S2. Appeared to cross-react with an HSV-1 protein) | Thermo Fisher Scientific | Cat# MA5-15253-HRP; RRID:AB_2537645 |  |
| V5 (used in all other figures) | BioLegend | Cat# 680602; RRID:AB_2566387 |  |
| Mouse IgG HRP | Cytiva | Cat# NA931; RRID:AB_772210 |  |
| Rabbit IgG HRP | Cell Signaling Technology | Cat# 7074; RRID:AB_2099233 |  |
| Bacterial and virus strains | | |  |
| HSV-1 (KOS strain) | ATCC | Cat# VR-1493 |  |
| HSV-1 ΔUL56 | Crump lab (ref. 30) | N/A |  |
| HSV-1 UL56-AAXA123 | Crump lab (ref. 30) | N/A |  |
| HSV-2 (strain 333) | Paludan lab (ref. 54) | N/A |  |
| VZV (ROka) | gift from Jeffrey Cohen (NIH, Bethesda, USA) | N/A |  |
| VACV (WR) | gift from Michael Way (The Francis Crick Institute, UK) | N/A |  |
| AdV-Cre-GFP | Vector Biolabs | Cat# 1700 |  |
| ZIKV (ZIKV/*H.sapiens*/Brazil/PE243/2015) | gift from Alain Kohl (University of Glasgow, UK) | N/A |  |
| IAV (PR8) | gift from Paul Digard (University of Edinburgh, UK) | N/A |  |
| Chemicals, peptides, and recombinant proteins | | | |
| LR Clonase™ II Plus enzyme | | Invitrogen | Cat# 12538120 |
| 2’3’-cGAMP | | Stratech | Cat# B8362-APE |
| bzATP | | Biotechne | Cat# 3312 |
| DCPIB | | Cayman | Cat# 34064 |
| A73004 | | Merck | Cat# 5083170001 |
| sulfasalazine | | Cayman | Cat# 15025 |
| YO-PRO1 | | Life Technologies | Cat# Y3603 |
| collagen | | Sigma | Cat# C8919 |
| violet live dead viability dye | | Thermo Fischer Scientific | Cat# L34955 |
| lipofectamine 2000 | | Thermo Fischer Scientific | Cat# 11668030 |
| chemiluminescent HRP substate ECL | | Perkin and Elmer | Cat# NEL104001EA |
| Critical commercial assays | | | |
| Pierce™ Silver Stain for Mass Spectrometry | | Thermo Fisher Scientific | Cat# 24600 |
| dual luciferase assay kit | | Promega | Cat# E1960 |
| Taqman universal PCR master mix | | Applied Biosystems | Cat# 4304437 |
| EXPRESS SYBR™ GreenER™ qPCR Supermix | | Thermo Fisher Scientific | Cat# 11784200 |
| Deposited data | | | |
| mass spectrometry data | | PRIDE | dataset identifier: PXD043229 |
| Experimental models: Cell lines | | | |
| HEK293 | | gift from Caetano Reis e Sousa (The Francis Crick Institute, UK) | N/A |
| HEK293T | | gift from Caetano Reis e Sousa (The Francis Crick Institute, UK) | N/A |
| HFF | | gift from Michael Weekes (University of Cambridge, UK) | N/A |
| HaCaT | | gift from Leonie Unterholzner (University of Lancaster, UK) | N/A |
| MeWo | | gift from Graham Ogg (University of Oxford, UK) | N/A |
| MEFs | | described in ref. 53 | N/A |
| Oligonucleotides | | | |
| *GAPDH* qPCR primer: 5’-CATGGCCTTCCGTGTTCCTA-3’ | | This study | N/A |
| *GAPDH* qPCR primer: 5’-CCTGCTTCACCACCTTCTTGAT-3’ | | This study | N/A |
| *UL56* qPCR primer: 5’-ACCAGCGACGAACGCAAAAC-3’ | | This study | N/A |
| *UL56* qPCR primer: 5’-ACCACCCCAAATACAGCATGGC-3’ | | This study | N/A |
| Recombinant DNA | | | |
| pCDNA3.2 | | Thermo Fischer Scientific | Cat# 12489019 |
| plenti6.3-V5 (BLAST) | | Thermo Fischer Scientific | Cat# V53306 |
| plenti6.3-V5 (PURO)  The *Blasticidin S deaminase* gene in plenti6.3-V5 (BLAST) was exchanged for the *PuroR* gene. | | This study. | N/A |
| lentiCRISPRv2 | | Gift from Andrew Basset (Wellcome Trust Sanger Institute, UK). Described in ref. 59. | N/A |
| pRL-TK | | Promega | Cat# E2241 |
| p125-Luc | | Kind gift from Takashi Fujita. Described in ref 62. | N/A |
| pcDNA3.2 STING | | This study. | N/A |
| pEXP103 GFP-UL56  Constitutively expresses the HSV-1 gene UL56 with a N terminal GFP tag. | | Described in ref 63. | N/A |
| pEXP103 GFP-UL6  Constitutively expresses the HSV-1 gene UL6 with a N terminal GFP tag. | | Described in ref 63. | N/A |
| p8.91 | | Gift from Greg Towers (University College London, UK). | N/A |
| pCMV-VSV-G | | Gift from Greg Towers (University College London, UK). | N/A |
| pNL4-3-deltaE-EGFP | | NIH AIDS reagents. Described in ref 56. | Cat# ARP-11100 |
| pHIV-2 ROD9 dEnvdNef GFP+ | | Gift from Nicholas Manel (Institut Curie, France). Described in ref 57. | N/A |
| plenti6.3 GFP-V5 (PURO)  Constitutively expresses GFP with a N-terminal V5 tag. | | This study. | N/A |
| plenti6.3 LRRC8A-V5 (PURO)  Constitutively expresses human LRRC8A with a C-terminal V5 tag. | | This study. | N/A |
| plenti6.3 LRRC8C-V5 (PURO)  Constitutively expresses human LRRC8C with a C-terminal V5 tag. | | This study. | N/A |
| plenti6.3 P2X7-V5 (PURO)  Constitutively expresses human P2X7 with a C-terminal V5 tag. | | This study. | N/A |
| plenti6.3 SLC19A1-V5 (PURO)  Constitutively expresses human SLC19A1 with a C-terminal V5 tag. | | This study. | N/A |
| plenti6.3 SLC46A2-V5 (PURO)  Constitutively expresses human SLC46A2 with a C-terminal V5 tag. | | This study. | N/A |
| plenti6.3 UL56-V5 (PURO)  Constitutively expresses untagged UL56 (due to presence of STOP codon). | | This study. | N/A |
| plenti6.3 UL56-AAXA1-V5 (PURO)  Constitutively expresses untagged UL56 (due to presence of STOP codon). PPXY motif 1 is mutated to AAXA. | | This study. | N/A |
| plenti6.3 UL56-AAXA2-V5 (PURO)  Constitutively expresses untagged UL56 (due to presence of STOP codon). PPXY motif 2 is mutated to AAXA. | | This study. | N/A |
| plenti6.3 UL56-AAXA3-V5 (PURO)  Constitutively expresses untagged UL56 (due to presence of STOP codon). PPXY motif 3 is mutated to AAXA. | | This study. | N/A |
| plenti6.3 UL56-AAXA123-V5 (PURO)  Constitutively expresses untagged UL56 (due to presence of STOP codon). PPXY motifs 1, 2 and 3 are mutated to AAXA. | | This study. | N/A |
| plenti6.3 GFP-V5 (BLAST)  Constitutively expresses human GFP with a C-terminal V5 tag. | | This study. | N/A |
| plenti6.3 P2X7-V5 (BLAST)  Constitutively expresses human P2X7 with a C-terminal V5 tag. | | This study. | N/A |
| plenti6.3 SLC46A2-V5 (BLAST)  Constitutively expresses human SLC46A2 with a C-terminal V5 tag. | | This study. | N/A |
| lentiCRISPR-v2 NT Guide-1  Expresses a non-targeting guide RNA (5’-ACGGAGGCTAAGCGTCGCAA-3’) bioinformatically predicted to not align with the human genome  and spCas9. | | Gift from Andrew Basset (Wellcome Trust Sanger Institute, UK). | N/A |
| lentiCRISPR-v2 NT Guide-2  Expresses a non-targeting guide RNA (5’-A CGCTTCCGCGGCCCGTTCAA -3’) bioinformatically predicted to not align with the human genome. | | Gift from Andrew Basset (Wellcome Trust Sanger Institute, UK). | N/A |
| lentiCRISPR-v2 LRRC8A Guide-1  Expresses a guide RNA (5’-A GGATCCTGAAGCCGTGGT -3’) targeting the *LRRC8A* gene. | | This study. | N/A |
| lentiCRISPR-v2 LRRC8A Guide-2  Expresses a guide RNA (5’-A GGCACCAGTACAACTACG -3’) targeting the *LRRC8A* gene. | | This study. | N/A |
| lentiCRISPR-v2 GOPC Guide-1  Expresses a guide RNA (5’-A GGAACATGGATACCCCGCCA -3’) targeting the *GOPC* gene. | | This study. | N/A |
| Software and algorithms | | | |
| GraphPad Prism v10 | | GraphPad Software | http://www.graphpad.com |
